# Transcriptome Analysis Reveals the Senescence Process Controlling the Flower Opening and Closure Rhythm in the Waterlilies (*Nymphaea* L.)

**DOI:** 10.3389/fpls.2021.701633

**Published:** 2021-10-04

**Authors:** Zhaoji Li, Weijuan Zhou, Peng Wang, Yanfu Chen, Shaojie Huo, Jian Wang, Daike Tian, Jun Niu, Ying Zhao, Xiqiang Song

**Affiliations:** ^1^Key Laboratory of Ministry of Education for Genetics and Germplasm Innovation of Tropical Special Trees and Ornamental Plants, Key Laboratory of Germplasm Resources Biology of Tropical Special Ornamental Plants of Hainan Province, College of Forestry, Hainan University, Haikou, China; ^2^Tropical Crops Genetic Resources Institute, Chinese Academy of Tropical Agricultural Sciences, Haikou, China; ^3^Shanghai Key Laboratory of Plant Functional Genomics and Resources, Shanghai Chenshan Plant Science Research Centre, Chinese Academy of Sciences, Shanghai Chenshan Botanical Garden, Shanghai, China

**Keywords:** flower opening and closure, flower senescence, gene regulation, cell signal transduction, cell metabolism, waterlily

## Abstract

Most waterlily flowers open at dawn and close after noon usually for three to four days, and thereafter wilt. The short lifespan of flowers restricts the development of the flower postharvest industry. The termination of flower movements is a key event during flower aging process. However, it is still unclear when the senescence process initiates and how it terminates the movement rhythm. In this study, we observed that the opening diameter of flowers was the smallest on the fourth (last) flowering day. Subsequent transcriptome profiles generated from petals at different flowering stages showed that the multiple signaling pathways were activated at the last closure stage (Time 3, T3) of the flowers, including Ca^2+^, reactive oxygen species and far red light signaling pathways, as well as auxin, ethylene and jasmonic acid signaling pathways. Moreover, In terms of cell metabolism regulation, the genes related to hydrolase (protease, phospholipase, nuclease) were upregulated at T3 stage, indicating that petals entered the senescence stage at that time; and the genes related to water transport and cell wall modification were also differentially regulated at T3 stage, which would affect the ability of cell expand and contract, and eventually lead to petal not open after the fourth day. Collectively, our data provided a new insight into the termination of flower opening in the waterlilies, and a global understanding of the senescence process of those opening-closure rhythm flowers.

## Introduction

*Nymphaea* L. (known as waterlily) belongs to the order of Nymphaeales, one of the most ancient angiosperm lineages. In the tree of plant life, Nymphaeales, Amborellales, and Austrobaileyales are the basal angiosperms, and recent genome study further confirmed that Nymphaeales and Amborellales are successive sister lineages to all other extant angiosperms (Qiu et al., 1999; Zeng et al., 2014; Zhang et al., 2020). As the globally popular ornamental plants, waterlilies have diverse colors and scents and are regarded as the national flower of Bangladesh and Sri Lanka (Chen et al., 2017; Zhang et al., 2020). In addition, waterlily flower also have economic and medicinal values due to their abundant metabolites and being good materials for essential oil extraction (Zhu et al., 2012; Zhao et al., 2019). However, the short lifespan of the flowers dampens their cut flower value and increases their transportation and storage cost. Thus, evaluating flower movements and senescence in *Nymphaea* will be highly useful in understanding the flowering characteristics of angiosperms and increasing their postharvest economic value.

In many ornamental plants, such as waterlilies, the flowers open and close for several days prior to petal wilting (Schneider and Chaney, 1981; van Doorn and van Meeteren, 2003; Povilus et al., 2015). After the final closing, the flowers begin to wilt, leading to unattractive to pollinators (van Doorn and van Meeteren, 2003; van Doorn, 1997). Prolonging the lifespan of cut flowers in these species requires not only the delay of the appearance of senescence symptoms but also the continuation of the flower movement rhythm. While, for other species that flower only once, if you want to prolong their lifespan, you can just concern about how to delay of the appearance of senescence symptoms. Although the regulation of flower movements and senescence has been extensively studied among flowering plants (van Doorn and van Meeteren, 2003; Rogers, 2013; Shahri and Tahir, 2013; van Doorn and Kamdee, 2014; Ahmad and Tahir, 2016), little is known about how the flower movement rhythm finishes and the role of senescence in this process.

Flower senescence is accompanied by the production and accumulation of reactive oxygen species (ROS), but the role of ROS in the initiation of flower senescence is still a subject of debate (van Doorn and Woltering, 2008). One recent study reported that ROS can act as a signal to trigger death via programmed or physiological cell death rather than by directly killing cells, and furthermore, that a basal level of ROS in cells is essential for normal cellular processes, as too much or too little can have negative effects on plant development (Mittler, 2016). Ca^2+^ is one of the most versatile signals involved in the control of cellular processes, including cell death (Ranty et al., 2016). The application of CaCl_2_ could prolong the vase life of cut flowers, and Ca^2+^ signaling-regulated genes, including *CaM*, *CBL1*, and *CBL3*, play an important role in this process (Zhang et al., 2018). Furthermore, the degradation of polymers such as proteins, lipids, and nucleic acids is also a vital event in the execution of cell death (Tripathi and Tuteja, 2007; Trivellini et al., 2016).

Hormones play important roles in various aspects of flower development, including flower senescence and flower movements. It is generally believed that ethylene and abscisic acid (ABA) trigger flower senescence, whereas cytokinins delay it, and the effects of auxins, gibberellic acid (GA), and jasmonates (JAs) in flower senescence are still inconclusive (Tripathi and Tuteja, 2007; Ahmad and Tahir, 2016). The functions of hormones in flower senescence vary among species; for example, ethylene can regulate senescence processes in ethylene-sensitive flowers but has little or no role in ethylene-insensitive flower systems (Ahmad and Tahir, 2016). Additionally, as plant growth regulators, hormones differentially affect flower opening depending on the species [summarized in van Doorn and van Meeteren (2003); van Doorn and Kamdee (2014)]. In roses, ethylene promotes or inhibits flower opening depending on the cultivar, which is due to differences in the sensitivity of the cultivar (Macnish et al., 2010; van Doorn and Kamdee, 2014). In waterlily, exogenous auxin promotes flower opening and suppresses flower closure, and the participation of auxin in the opening-closing process has been further unveiled (Ke et al., 2018). Thus, hormones may play complex and diverse roles during flower development in a species-dependent manner.

Flower opening and closure rhythms have been reported to be controlled by a dual system: light and circadian clocks. The intrinsic flower movement rhythms regulated by the circadian clock are influenced by light signal appearance and disappearance (Bai and Kawabata, 2015). The mechanism of flower circadian movements is considered to be related to the reversible expansion and shrinkage of cells (van Doorn and van Meeteren, 2003; Ke et al., 2018). In waterlily, cell wall remodeling is essential for cell shape changes, which involve the differential regulation of cellulose synthase (CesA), expansin (EXP), and xyloglucan endotransglucosylase (XTH/XET) (Ke et al., 2018). In addition, water flow, as mediated by aquaporins that act as transmembrane proteins, is also vital for cell expansion (Ma et al., 2008; van Doorn and Kamdee, 2014). Notably, the above processes, including light signaling, cell wall modification, and water relations, are involved in not only flower movements but also flower senescence. *Phototropin1* (*PHOT1*), *chlorophyll a-b binding protein* (*LHCB*), and other light signaling-related genes are involved in the senescence process of ephemeral hibiscus flowers (Trivellini et al., 2016). Cell wall degradation and water loss are regarded as vital events in the progression of flower senescence (Eason et al., 1997; Elanchezhian and Srivastava, 2001; Shahri and Tahir, 2013). What’s more, flower opening and closure rhythms and flower senescence are both under the regulation of the biological circadian clock (van Doorn and van Meeteren, 2003; Xu et al., 2007; van Doorn and Kamdee, 2014). Therefore, the rhythm of flower opening and closure has something in common with the progression of flower senescence, which means that the flower rhythm is easily affected by the aging process.

In this study, we recorded the floral movement rhythms of waterlily flowers. Transcriptome analysis involving cellular signal and metabolism processes of the petals at three flowering stages provided a global indication that gene networks coordinate the abolishment of petal movement rhythms and the initiation of senescence. This study offers an integrated approach for understanding the interconnections between flower circadian movements and flower senescence, providing useful information for studying similar traits in flowering plants across angiosperms.

## Materials and Methods

### Plant Materials and Growth Condition

*Nymphaea* ‘Blue Bird’ (R. Sawyer, 1927, website^1^) plants were cultivated in outdoor pots at Hainan University, Haikou. The flowers on the first blooming day were harvested during 8:30 am–9:30 am, and the cut flowers were immediately hydroponically grown in a chamber controlled at 25°C with a 12-h light/12-h dark photoperiod (7:00 am–19:00 pm light/19:00 pm–7:00 am dark).

### Time-Lapse Photography and Measurement of Flowering Diameter

**FIGURE 1:**
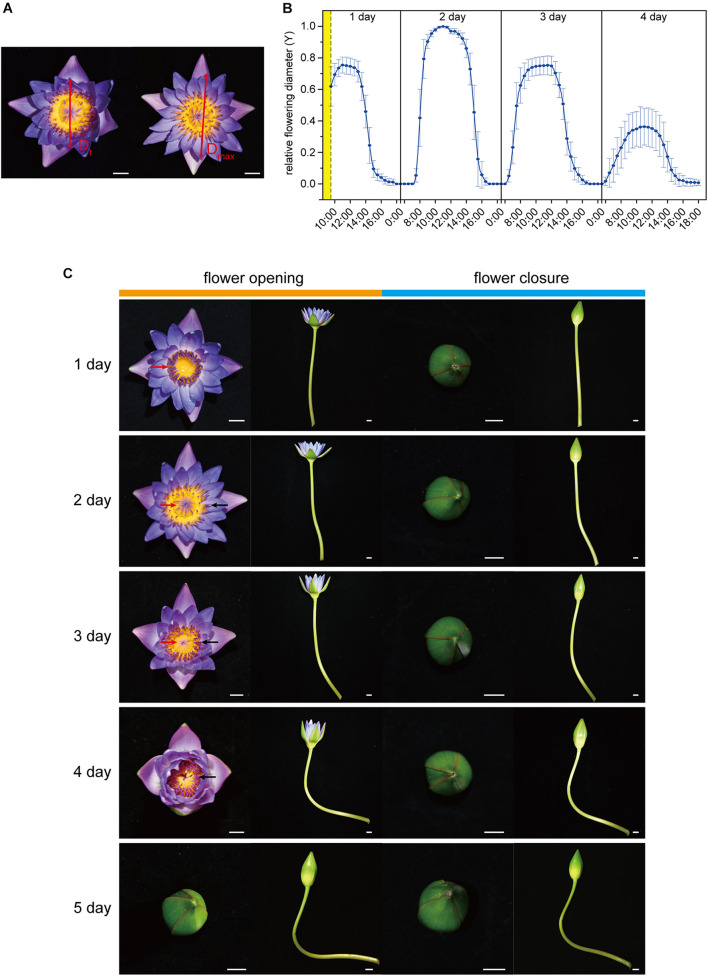
Flower opening and closure process. **(A)** The flower opening and closing process was recorded based on the movement of a couple of petals in an opposite position. The measured diameter at time t (D_t_) was normalized as Y = D_t_/D_max_, where D_max_ represents the maximum of D_t_. **(B)** Flower opening and closure pattern in 1–4 days. The relative flowering diameter (Y) was measured and calculated every half hour. The cut flowers were harvested from 8:30 am to 9:30 am in the morning of the first blooming day (yellow part). As the flowers were closed from 17:30 pm to 6:30 am (Y = 0), data only at 17:30, 00:00, and 6:30 are shown, and the remaining data from this period have been omitted. **(C)** Opening and closing of waterlily flower over 1–5 days. The opening was captured at around 11:00 am, and the closure was captured at around 17:00 pm daily. The red arrows and black arrows mark the immature and mature stamens, respectively. Scale bar, 1 cm **(A,C)**.

Digital cameras (D750, Nikon, Japan) were fixed on the top of the flowers such that the position of the petals could be seen clearly, and flower images with an overhead view were taken automatically at 30-min intervals for five consecutive days. Diameters (D, Figure 1A) of flowers during the opening and closure process were measured using ImageJ. Then, the normalized relative flowering diameter (Y) was calculated as,


Y=DDt/max

In the equation, D_t_ represents D at time t, and D_max_ is the maximum of D during the five consecutive days for each flower. After the calculation of Y values for each flower, the average Y value from no less than six independent flowers was calculated for each time point.

### RNA-Seq, Expression Annotation, Gene Ontology (GO), Kyoto Encyclopedia of Genes and Genomes (KEGG) Pathway Enrichment and Gene Expression Analyses

The petal samples were collected at three flowering times: 11:00 am on the second day (flower opening), 17:00 pm on the second day (flower temporary closure), and 17:00 pm on the fourth day (flower final closure). Each sample was collected from at least three flowers, and the samples at each time point with three replicates were collected. All the collected petal samples were immediately frozen in liquid nitrogen and stored at −80°C for RNA extraction. The total RNA of the waterlily flowers was extracted using the RNAprep Pure Plant Kit (Tiangen). Nine RNA-Seq libraries (Each time point with tree replicates) were sequenced on an Illumina HiSeqTM2000 platform. After removing the adapter sequences, low quality sequences, unknown reads (N percentage > 5%), and low-quality reads, the remainder of the raw reads (clean reads) were obtained. Next, the clean reads were used for transcriptome *de novo* assembly using Trinity (v2.4.0). The unigenes were then obtained by removing the redundancy and further splicing the reads using Tgicl (v5.18.2). These unigenes were annotated using the BLASTx alignment (E-value ≤ 10^–5^) to various protein databases, including the NCBI Non-redundant (Nr), Swiss-Prot, and KEGG databases. Unigene expression abundance differences among the samples were represented using the Fragments Per Kilobase of transcript per Million mapped reads (FPKM) method. Differential expression analysis among different stages was performed with kallisto (v0.43.1) and edgeR (v3.16.5). Genes with a fold change > 2 and false discovery rate (FDR) ≤ 0.001 were assigned as differentially expressed genes (DEGs). KEGG^2^ was used to perform pathway analysis of the DEGs. Statistical enrichment of the DEGs for the GO terms was implemented using ClusterProfiler (v3.2.0). Significantly enriched GO terms and KEGG pathways (q-value ≤ 0.05) were identified based on a hypergeometric test.

In order to furtherly investigate the genes regulation of petal cell at T3 stage, we analyzed the genes involved in the vital signaling pathways including Ca^2+^, ROS, light and hormone signaling pathway. And then, we investigated the gene regulation involved in cellular metabolism which involved proteins, lipids and nucleic acids degradation, water transportation and cell wall modification. By analyzing the two aspects, signal transduction and substance metabolism, of petal cell, we aimed to evaluate the growth condition of petals at T3 stage and answer the question “why waterlily petals do not reopen after the fourth flowering day?” And the heatmap of gene expression were drawn using TBtools (Chen et al., 2018).

### qRT-PCR Assay

To verify the results from RNA-Seq, we selected a total 13 unigenes for quantitative real-time PCR (qRT-PCR) analysis. Primer sequences designed with Primer Premier 5.0 software are shown in Supplementary Table 1. cDNA synthesis and qRT-PCR were performed using HiScript III RT SuperMix for qPCR (+ gDNA wiper) and ChamQ Universal SYBR qPCR Master Mix (Vazyme, Biotech (Nanjing) Co., Ltd., China). Actin 11 was selected as an internal control (Luo et al., 2010). The experiments were performed on ABI QuantStudio 1 Real-Time PCR System (Applied Biosystems, Waltham, MA, United States). Three technical replicates were performed for qRT-PCR. The 2^–△△CT^ method (Schmittgen and Livak, 2008) was used for the calculation of relative expression level of the unigenes.

## Results

### Observation of Flower Movement in Waterlily

The cut flowers of *Nymphaea* ‘Blue Bird’ opened and closed successively for four days and no longer reopened on the fifth day (Figures 1B,C; Supplementary Figure 1). They were open from 7:00 to 10:00 and closed from 12:00 to 16:00. On the second blooming day, the openness of the flowers was the largest, and the maximum value of Y reached 1, while on the fourth day, the openness was the smallest, and the highest Y value was no more than 0.4. The openness of the first day and the third day was similar, with a maximum Y value of more than 0.7 (Figure 1B). The flowers did not open from the fifth day. waterlily flowers are dichogamous and protogynous. The first blooming day is the female phase, and at this stage the stamens were immature and standing erect, and the stigma disk was exposed and secreted stigmatic fluid. The male phase began from the second day, at which stage the stamens covered the top of the stigmatic disk and gradually opened and matured from the outside to the inside until the fourth day, and all the stamens had matured in the fourth day (Figure 1C). It is worth noting that although there were no visible senescence symptoms, such as wilting, abscising, or fading, on the petal on the fourth day, the smaller openness indicated that the flower probably had started aging, and the opening and closing rhythm had become weaker.

### Transcriptome Profiling of the Waterlily Flower at Three Flowering Stages

**FIGURE 2:**
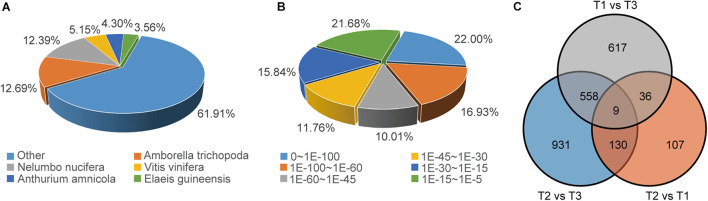
Overview of the unigene annotations and DEG numbers. **(A)** Species distribution of NCBI NR annotation of unigenes. **(B)** E-value distribution of NCBI NR annotation of unigenes. **(C)** Venn diagram of DEGs at three flowering stages.

In order to further elucidate the mechanism of petal movement and senescence, we performed a *de novo* RNA-Seq analysis based on the three key time points of flowering. Samples at 11:00 am (T1) on the second flowering day were used as the control when the flowers were opening, and samples at 17:00 pm (T2) on the second flowering day and at 17:00 pm (T3) on the fourth day represented the temporary flower closure stage (also the middle of the flower circadian movement) and the final flower closure stage (also the end of the flower circadian movement), respectively. After assembly and removal of redundancy, 136,095 unigenes were obtained, among which the median length of unigenes was 1,393 nt and the value of N50 was 2,479 nt. For annotation, 133,841 unigenes could be annotated to the NCBI NR database, while 103,413 and 104,705 unigenes could be annotated to the KEGG and Swiss-Prot databases, respectively. According to the NCBI NR annotation and E-value distribution, 48.94% of the annotated sequences had strong homology (E-value < 10^–45^). The five top-hit species based on NCBI NR annotations included *Amborella trichopoda, Nelumbo nucifera, Vitis vinifera, Anthurium amnicola*, and *Elaeis guineensis* (Figures 2A,B). Differences in gene expression at three stages during flowering were examined, and DEGs were identified by pairwise comparisons of the three libraries. Comparisons of the three stages identified 282(up: 129; down: 153), 1,628(up: 705; down: 923), and 1,220(up: 365; down: 855) DEGs in pairs of T2 vs. T1, T2 vs. T3, and T1 vs. T3 (Figure 2C, Supplementary Figure 2). According to GO enrichment and KEGG pathway analysis, in the T2 vs. T1 comparison group, cellular glucan metabolic process, glucan biosynthetic process, N-glycan biosynthesis pathway, etc., were enriched, which suggested the petal movements in second flowering day mainly involved the processes of glycan synthesis and metabolism (Supplementary Figure 3). In T2 vs. T3 comparison group, oxidoreduction coenzyme metabolic process, cellular amino acid biosynthetic process, oxidative phosphorylation pathway, etc., were enriched (Supplementary Figure 3), which suggested the cellular foundational processes involving amino acid biosynthesis and Adenosine triphosphate (ATP) generation had a great change at T3 stage, and these supported that the senescence process had been initiated since T3 stage in waterlily petal.

### Gene Regulation Involved in Cell Signal Transduction of Waterlily Petals

#### Ca^2+^ Signaling

**FIGURE 3:**
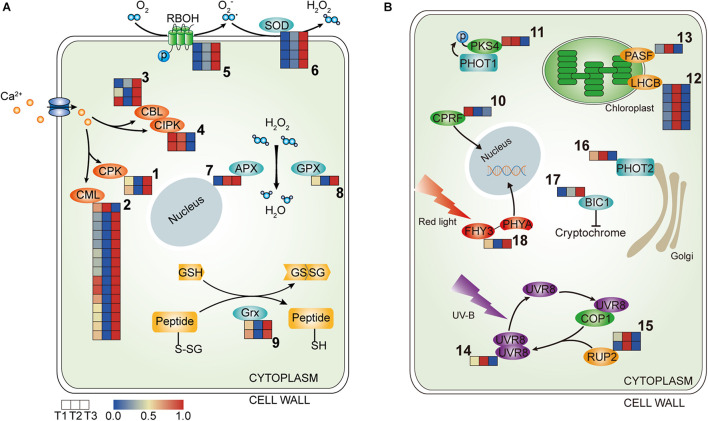
Heatmap of the components involved in the signal transduction in the three petal transcriptomes, T1, T2, and T3. **(A)** The expressed genes assigned to Ca^2+^ signaling and ROS signaling. **(B)** The expressed genes assigned to light signaling (the corresponding genes are listed in Tables 1, 2 according to the labeled number “1” to “18” in each map). Red and blue colors indicate upregulated and downregulated transcripts, respectively.

Ca^2+^ signatures are sensed and decoded by Ca^2+^ sensor proteins. These include calmodulin-like proteins (CMLs), Ca^2+^-dependent protein kinases (CPKs), calcineurin B-like proteins (CBLs), and CBL-interacting kinases (CIPKs), which together form intricate signaling networks for translating Ca^2+^ signatures into downstream responses (Batistič and Kudla, 2012). In the transcriptome profile, *CPKs*,*CBLs* and almost all *CML* homologs were upregulated at T3, while only homologs of *CIPK5* maintained high transcript levels at T1 and T2 (Figure 3A and Table 1), suggesting that Ca^2+^ signaling were more active at T3 in waterlily petal cell.

**TABLE 1:** Ca^2+^ signaling and ROS signaling (corresponding to Figure 3A).

Heatmap number	Gene ID	Best	FPKM	FPKM	FPKM
		homolog	(T1)	(T2)	(T3)
1	cluster_contig27576_	CPK2	71.3	35.6	138.5
	cluster_contig39366_	CPK33	10.3	1	32.3
2	TRINITY_DN60921_c0_g2_i4	CML49	118.1	192.3	37.3
	TRINITY_DN52238_c0_g1_i1	CML45	29.4	16.5	129.6
	cluster_contig35093_	CML10	234.6	165.6	658.9
	TRINITY_DN69672_c1_g4_i1	CML42	59.5	34.8	267.5
	TRINITY_DN52238_c0_g1_i2	CML45	19.6	9.2	157.4
	cluster_contig23956_	CML36	1131.6	640.1	2880.7
	cluster_contig25498_	CML42	73.5	44.8	189.3
	TRINITY_DN55942_c0_g2_i2	CML41	133	69.7	423.1
	cluster_contig3666_	CML49	44.6	3.1	60.8
	TRINITY_DN61845_c1_g1_i10	CML49	37.1	1.3	50.1
	cluster_contig36423_	CML28	128	26.3	261
	cluster_contig48502_	CML8	705	308.3	1549
	TRINITY_DN55942_c0_g2_i1	CML41	169	59.3	531.3
	TRINITY_DN52629_c0_g1_i1	CML23	737.4	245.8	1775
	TRINITY_DN52629_c0_g1_i2	CML23	809.9	221.5	1880.7
3	TRINITY_DN65373_c0_g2_i6	CBL1	13.7	27	141.2
	cluster_contig20023_	CBL9	3.6	0.3	26
	TRINITY_DN69242_c0_g1_i12	CBL9	54.4	1.3	56.7
4	TRINITY_DN64277_c0_g1_i2	CIPK5	212	168.1	37.8
	cluster_contig34530_	CIPK5	417.3	376.6	86.6
5	cluster_contig25570_	RBOHD	259.6	512.3	3154.6
	cluster_contig25571_	RBOHB	92.4	161.1	896.8
	TRINITY_DN52186_c0_g1_i1	RBOHC	193.3	334	2198.7
6	cluster_contig25339_	SOD	14.9	25.6	158.6
	TRINITY_DN65286_c0_g1_i13	SOD	148.5	198.6	759.8
	TRINITY_DN65286_c0_g1_i14	SOD1	39.2	52.1	278.9
	TRINITY_DN65286_c0_g1_i11	SOD1	16.5	22.5	124.2
7	TRINITY_DN56835_c0_g1_i6	APX1	205.5	825.8	891.5
8	TRINITY_DN69062_c2_g1_i2	GPX6	5.1	0	30.4
9	cluster_contig23012_	GrxC2	59.4	18.8	139.8
	TRINITY_DN62533_c0_g1_i3	Grx	36.5	3.9	72.7

#### ROS Signaling

ROS homeostasis is mediated by ROS-producing and ROS-scavenging genes. NADPH oxidases, termed respiratory burst oxidase homologs (RBOHs), account for the generation of apoplastic ROS (Mittler, 2016). Gene transcripts encoding *RBOH* were more abundant at T3, suggesting that ROS were more generated in the waterlily petals at the last closure stage (Figure 3A and Table 1). Meanwhile, transcripts encoding enzymes committed to ROS scavenging were also higher in abundance at T3, including *Superoxide dismutase* (*SOD*), *Ascorbate peroxidase* (*APX*), and G*lutathione peroxidase* (*GPX*), suggesting the existence of a petal active ROS-scavenging system to dispose of ROS at the last closure stage and contribute to the modulation of ROS signaling (Figure 3A and Table 1). Moreover, thiol can be reduced by glutaredoxin (Grx), in the transcriptome profiles, *Grxs* were also highly expressed at T3 (Figure 3A and Table 1). Overall, gene assigned to ROS-producing and ROS-scavenging were both upregulated at T3 stage.

#### Light Signaling

Genes related to light signaling were differentially regulated among various stages in the waterlily flowers. *Light-inducible protein CPRF2*, a G-box-binding transcription factor (TF), was highly expressed at T1 (Figure 3B and Table 2). Phytochrome Kinase Substrate 4 (PKS4) is an element of Phototropin-1 (PHOT1) signaling and constitutes a molecular link between PHOT1 and phytochrome signaling, as its phosphorylation status is controlled by the combined activities of PHOT1 and phytochromes (Demarsy et al., 2012). The *PKS4* homolog was upregulated at both T1 and T2, implying that the circadian flower movements on the second day were accompanied by light signaling transduction (Figure 3B and Table 2). Moreover, genes involved in photosynthesis were additionally induced at T2, including *light-harvesting chlorophyll a-b binding protein* (*LHCB*) and *Photosystem I reaction center subunit III* (*PASF*), suggesting that the photosystems responded to changes in light quantity during flower closure on the second day; however, both genes were downregulated at T3, implying that a similar photosynthetic reaction was not required at the final closure stage (Figure 3B and Table 2).

**TABLE 2:** Light signaling (corresponding to Figure 3B).

Heatmap number	Gene ID	Best homolog	FPKM (T1)	FPKM (T2)	FPKM (T3)
10	TRINITY_DN70167_c0_g1_i2	CPRF2	145.7	29.0	37.4
11	TRINITY_DN57183_c0_g1_i2	PKS4	193.0	232.7	20.3
12	cluster_contig22181_	LHCB	6.3	607.5	3.7
	TRINITY_DN52716_c1_g1_i1	LHCB8	60.0	545.8	47.0
	cluster_contig45520_	LHCB8	131.3	695.8	96.7
	cluster_contig48182_	LHCB2.1	94.3	491.3	63.7
	TRINITY_DN54053_c0_g1_i1	LHCB5	110.1	594.0	87.2
13	TRINITY_DN57338_c5_g1_i2	PSAF	158.0	813.0	91.0
14	cluster_contig11070_	UVR8	2007.3	6056.9	674.1
15	TRINITY_DN51849_c0_g1_i3	RUP2	77.7	284.5	30.7
	TRINITY_DN51849_c0_g1_i4	RUP2	175.3	718.6	162.3
16	cluster_contig32122_	PHOT2	61.7	143.4	12.2
17	cluster_contig29762_	BIC1	301.3	556.0	1945.4
18	TRINITY_DN63136_c2_g1_i12	FHY3	11.0	1.7	34.9

Plants use the UV-B photoreceptor UVR8 to perceive UV-B, which can induce a multitude of physiological responses influencing the growth and development of plants. The UVR8 homodimer, which is an inactive form, can be monomerized by UV-B absorption, following which the UVR8 monomer interacts directly with COP1 to initiate UV-B signaling. The UVR8 monomer also can be redimerized through the action of REPRESSOR OF UV-B PHOTOMORPHOGENESIS (RUP, WD40-repeat proteins), which disrupts the UVR8-COP1 interaction, regenerates the UVR8 homodimer, and impinges on UVR8 function (Gruber et al., 2010; Rizzini et al., 2011; Tilbrook et al., 2013). In the transcriptome profiles, *UVR8* and *RUP2* were highly expressed at T2, implying that temporary flower closure triggered genes associated with UV-B, and the regeneration of the UVR8 homodimer catalyzed by RUP might serve as a storage of UVR8 for flower reuse the following day, while the storage disappears on the final day of flower closure (Figure 3B and Table 2). Phototropins (PHOT1 and PHOT2 in *Arabidopsis*) are blue light receptors. The expression of *PHOT2* was downregulated at T3 (Figure 3B and Table 2). In contrast, *BLUE-LIGHT INHIBITOR OF CRYPTOCHROMES 1* (*BIC1*) was activated at T3, suggesting that blue light signaling was suppressed at the last closure stage (Figure 3B and Table 2). What’s more, *FAR-RED ELONGATED HYPOCOTYL3* (*FHY3*), a TF involved in far-red light signaling, was upregulated at T3, (Figure 3B and Table 2). Hence, it appears that blue light (including UV-B) and far-red signaling participate in floral temporary closure and final closure, respectively, suggesting the distinct roles of blue light and far-red light in the regulation of flower circadian movements and senescence.

#### Hormone Coordination

**FIGURE 4:**
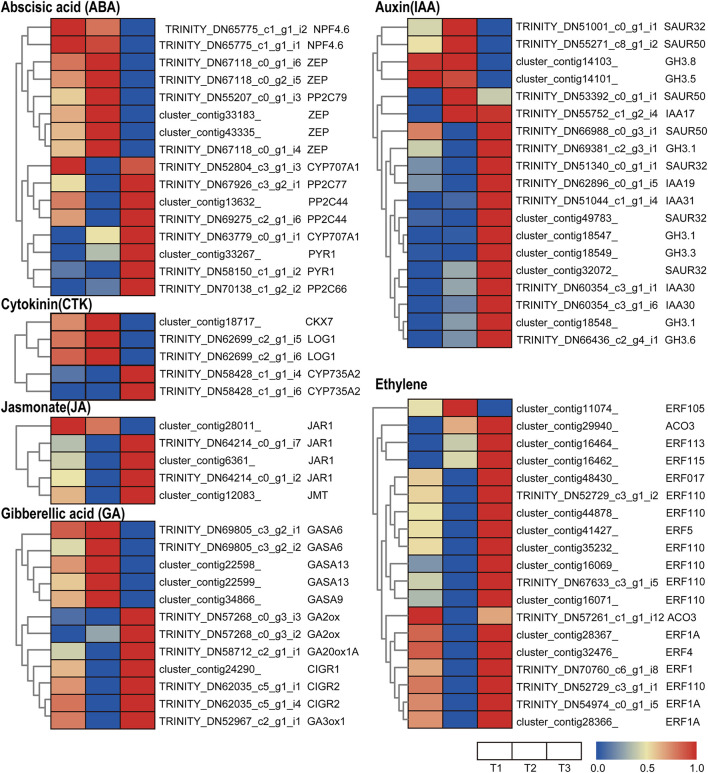
Heatmap of the expressed genes assigned to hormone signal transduction pathways in the three petal transcriptomes, T1, T2, and T3. Red and blue colors indicate upregulated and downregulated transcripts, respectively.

Genes related to auxin signaling and synthesis were differentially regulated in various flowering stages in waterlily. Most early auxin response genes are classified into three families: SAURs, AUX/IAAs, and GRETCHEN HAGEN3s (GH3s) (Hagen and Guilfoyle, 2002). The small auxin up RNA (SAUR) family represents the largest family of early auxin response genes. The DEGs of SAURs in the transcriptome profile show that three *SAUR* homologs were induced at T2, while four *SAUR* homologs were stimulated at T3, suggesting that an early auxin response occurred in both types of flower closure (Figure 4). Acting as transcriptional repressors, Aux/IAAs have a vital role in nuclear auxin signaling. Free IAA can be catalyzed to IAA amide conjugates by the Gretchen Hagen3 (GH3) family of auxin-inducible acyl amido synthetases (Staswick et al., 2005). *IAA17*, *IAA30*, and *IAA19* homologs were highly expressed at T3, implying that auxin signaling was more active at that time (Figure 4). Five *GH3* homologs were upregulated at T3, while *GH3.5* and *GH3.8* were upregulated at T1 and T2, suggesting that different *GH3* members displayed distinct responsiveness to the three flowering stages (Figure 4).

JASMONATE RESISTANT 1 (JAR1) encodes an enzyme catalyzing the conjugation of (+)-7-iso-JA with l-Ile, which is the endogenous bioactive jasmonate (JA-Ile) (Fonseca et al., 2009). Jasmonate O-methyltransferase (JMT) catalyzes the methylation of jasmonate into Methyl jasmonate (MeJA). MeJA acts as an airborne signal that mediates interplant communication for defense responses (Seo et al., 2001). In the transcriptome profiles, *JMT* and three out of four *JAR1* homologs were upregulated at the T3 stage, implying that the last closure movement involved JA synthesis (Figure 4).

*Trans*-zeatin and isopentenyladenine-type cytokinins were thought to be the predominant cytokinins. Several genes involved in cytokinin (CTK) metabolism were identified in the waterlily flower. CYP735A1 and CYP735A2 are cytochrome P450 monooxygenases (P450s) that catalyze the biosynthesis of *trans-*zeatin (Takei et al., 2004). The transcriptome profile shows that *CYP735A2* homologs were upregulated at T3 (Figure 4), suggesting *trans-*zeatin was synthesis at the last closure stage. While another CTK biosynthesis gene, *LONELY GUY* (*LOG1*), was upregulated at T2 (Figure 4), and the expression pattern of the cytokinin-degrading enzyme *cytokinin dehydrogenase* (*CKX*) is in line with *LOG1* (Figure 4), which suggested the CTK content is precisely controlled when the flower is temporarily closed.

Several genes related to abscisic acid (ABA) synthesis, signaling, and metabolism were differentially regulated at various flowering stages. Zeaxanthin epoxidase (ZEP) converts zeaxanthin into violaxanthin via antheraxanthin and plays an important role in ABA biosynthesis (Endo et al., 2014). NPF4.6 is a member of the NITRATE TRANSPORTER1/PEPTIDE TRANSPORTER FAMILY (NPF) and mediates ABA uptake into cells (Kanno et al., 2012). In the transcriptome profiles, *ZEP* and *NPF4.6* were highly expressed at the T1 and T2 stage, suggesting that the floral opening and closure movement was accompanied by ABA biosynthesis and transport (Figure 4). The core ABA signaling components are composed of soluble pyrabactin resistance 1/PYR1-like/regulatory component of ABA receptor (PYR/PYL) ABA receptors, clade A type 2C protein phosphatases (PP2Cs), and group III subfamily 2 Snf1-related kinases (SnRK2s) (Umezawa et al., 2009). The transcriptome profiles show that *PYR* and most of the *PP2C* homologs were upregulated at T3, suggesting that ABA signaling is active at that time (Figure 4). However, *CYP707A*, catalyzing ABA 8’-hydroxylation, which results in ABA degradation (Kushiro et al., 2004), was upregulated at T3 (Figure 4). Altogether, the expression of genes involved in ABA synthesis and signaling does not appear to be synchronous, suggesting the complex regulation of the ABA pathway during flower development.

Ethylene is involved in a number of essential processes during flower development, especially in petal senescence. 1-aminocyclopropane-1-carboxylic acid (ACC) converted into ethylene is catalyzed by the 1-aminocyclopropane-1-carboxylic oxidase (ACO). In the transcriptome profile, *ACO* homologs were more abundant at T3, implying that ethylene was synthesized at the last floral closure stage (Figure 4). Additionally, the ethylene-responsive factor (ERF) family of TFs was found to be differentially expressed during the floral opening-closure process: almost all *ERF* homologs were upregulated at T3, except for *ERF105*, which was stimulated at T2 (Figure 4). In brief, *ACOs* and most of the *ERFs* were activated at T3, indicating that the final closure of waterlily was associated with ethylene biosynthesis and signaling, which could activate downstream genes involved in cell wall modification and cell senescence.

GA20ox and GA3ox are key enzymes in gibberellin (GA) biosynthesis and catalyze the hydroxylation steps of the ent-gibberellane skeleton. GA2ox can catalyze bioactive GA into inactive forms. The expression of *GA3ox* was stimulated at T1 and T3, and *GA20ox* was more highly expressed at T3, implying that flower opening and last closure involved GA synthesis (Figure 4). Moreover, *GA2ox*, which has an inactive GA function, was also upregulated at T3, suggesting that the effect of the GA was impeded by subsequent deactivation at the last closure time (Figure 4). The Gibberellic Acid-Stimulated Arabidopsis (GASA) peptide family, which comprises a portion of the cysteine-rich peptides (CRPs), is vital for plant growth and development (Silverstein et al., 2007). Chitin-inducible gibberellin-responsive (CIGR) are GRAS proteins from the PAT1 branch and play key transcriptional regulatory roles in plant development and defense. The role of GASA and CIGR proteins in flower development is not yet clear. Interestingly, homologs of *GASA* and *CIGR* were downregulated at T3 and T2, respectively, implying the distinct responses of GA signaling to the different flower closure times (Figure 4).

In summary, at the different flowering stages, petals require specific hormone coordination to accomplish the relevant cell activity. At T1 stage, ABA, GA, CTK and ethylene signaling showed active; at T2 stage, ABA and CTK signaling were induced; while at the last closure period (T3), there appeared to be more intensive auxin, JA and ethylene signaling in petals, and the active ethylene signaling might lead to petal senescence and finishing of the movements.

### Gene Regulation Involved in Cell Metabolism of Waterlily Petals

#### Degradation of Biomacromolecule

**FIGURE 5:**
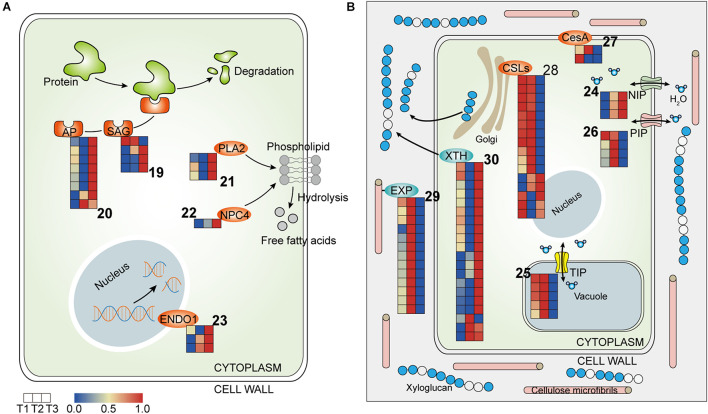
Heatmap of components involved in cell metabolism in the three petal transcriptomes, T1, T2, and T3. **(A)** The expressed genes assigned to hydrolases. **(B)** The expressed genes assigned to water relation and cell wall modification (the corresponding genes are listed in Tables 3, 4 according to the labeled number “19” to “30” in each map). Red and blue colors indicate upregulated and downregulated transcripts, respectively.

Protein degradation has a prime role in flower senescence and is involved in different types of proteases, including cysteine proteases, serine proteases, and aspartic proteases. Several *senescence-associated genes* (*SAGs*), including genes encoding cysteine proteases (*SAG39*) and aspartyl proteases (*AP*), were identified and highly expressed at T3 (Figure 5A and Table 3), suggesting the degradation of protein, which is indicative of the senescence that was triggered at the final closure stage. Phospholipids, as components of membranes, can be degraded by phospholipase. Homologs of *phospholipase A2* (*PLA2*) and *non-specific phospholipase C4* (*NPC4*) were stimulated at T3, implying that the membrane structure was damaged at the final closure stage (Figure 5B and Table 3). Moreover, the transcript levels of *Endonucleases 1* (*ENDO1*) were also abundant at T3, suggesting that the hydrolysis of DNA/RNA was active at that time (Figure 5A and Table 3). Overall, these results suggested an irreversible senescence process that involved the activation of proteases, phospholipases, and nucleases that were initiated from flower closure on the fourth day.

**TABLE 3:** Hydrolases (corresponding to Figure 5A).

Heatmap number	Gene ID	Best homolog	FPKM (T1)	FPKM (T2)	FPKM (T3)
19	TRINITY_DN68774_c1_g1_i5	SAG101	48.9	43.9	3.2
	cluster_contig48875_	SAG102	94.0	258.7	458.3
	TRINITY_DN55038_c0_g1_i3	SAG39	27.0	31.3	509.1
	cluster_contig42152_	SAG39	32.4	37.7	714.9
20	TRINITY_DN70240_c0_g2_i4	At4g16563	90.0	65.2	398.3
	TRINITY_DN70240_c0_g1_i1	At4g16563	2334.5	701.2	6841.6
	TRINITY_DN70240_c0_g2_i2	At4g16563	626.3	163.1	1834.9
	TRINITY_DN62694_c0_g1_i3	APF2	248.5	103.5	777.0
	TRINITY_DN62694_c0_g1_i5	APF2	258.2	127.2	897.2
	TRINITY_DN62694_c0_g1_i6	APF2	3.9	0.7	31.2
	TRINITY_DN52882_c0_g1_i1	At5g10770	64.7	219.0	495.0
	TRINITY_DN54216_c4_g1_i1	At5g10770	26.8	542.5	229.2
21	TRINITY_DN56115_c0_g1_i7	PLA2-III	0.5	0.0	29.4
	cluster_contig33557_	PLA2-A LPHA	15.0	1.7	48.2
	TRINITY_DN56115_c0_g1_i3	PLA2-III	57.7	7.3	512.1
22	cluster_contig35501_	NPC4	71.9	145.7	919.3
23	TRINITY_DN54764_c3_g1_i6	ENDO1	172.6	70.8	458.3
	TRINITY_DN54764_c3_g1_i2	ENDO1	0.0	12.4	45.5
	cluster_contig23990_	ENDO1	0.0	38.9	89.0

#### Water Transportation

Aquaporins (APQs), which control water transport in cells, have been implicated in many developmental processes in plants, including cell expansion, organ movement, and elongation (Tyerman et al., 2002). APQs can be classified in several subfamilies, including plasma membrane intrinsic proteins (PIPs), tonoplast intrinsic proteins (TIPs), NOD26-like intrinsic proteins (NIPs), etc., (Danielson and Johanson, 2008). In the transcriptome profiles, *NIP1-1* was more highly expressed at T2 and T3, while *TIPs* and *PIPs* were upregulated at T2 and were downregulated at T3, suggesting that water flow was more frequent and complex at T2 than at T3 (Figure 5B and Table 4).

**TABLE 4:** Water relation and cell wall modification (corresponding to Figure 5B).

Heatmap number	Gene ID	Best homolog	FPKM (T1)	FPKM (T2)	FPKM (T3)
24	TRINITY_DN53101_c5_g1_i4	NIP1-1	13.5	90.0	300.2
	cluster_contig28870_	NIP1-1	6.0	48.7	122.9
	TRINITY_DN53101_c5_g1_i2	NIP1-1	26.1	263.3	651.0
25	TRINITY_DN64189_c1_g1_i6	TIP1-1	3548.6	3996.9	174.9
	TRINITY_DN58925_c4_g1_i3	TIP2-1	381.9	525.4	19.3
	cluster_contig35170_	TIP1-1	462.8	628.7	34.9
	TRINITY_DN70478_c2_g1_i8	TIP4-1	328.7	614.2	78.1
	cluster_contig48146_	TIP4-1	61.6	149.6	21.0
26	TRINITY_DN57533_c3_g2_i3	PIP2-1	212.4	169.7	43.3
	TRINITY_DN53385_c1_g4_i1	PIP2-6	50.7	236.3	13.3
	TRINITY_DN66083_c2_g2_i1	PIP2-1	76.5	236.2	39.9
	cluster_contig32351_	PIP2-5	215.4	677.2	119.5
27	TRINITY_DN58846_c0_g1_i7	CESA8	19.7	59.0	3.9
	TRINITY_DN70606_c3_g1_i9	CESA1	42.1	0.0	0.0
28	TRINITY_DN53435_c1_g1_i9	CSLE6	1191.6	1104.7	191.9
	TRINITY_DN53435_c1_g1_i6	CSLE1	368.1	346.1	85.6
	cluster_contig14537_	CSLE2	1109.8	975.4	177.0
	cluster_contig35632_	CSLE1	1396.0	1238.3	318.3
	cluster_contig14532_	CSLE6	61.3	72.5	8.1
	TRINITY_DN53435_c1_g1_i8	CSLE6	4953.2	5611.6	1015.8
	TRINITY_DN53435_c1_g1_i3	CSLE6	248.4	277.9	11.7
	cluster_contig14536_	CSLE6	2270.6	2270.1	451.9
	TRINITY_DN67870_c2_g2_i4	CSLC5	8.5	22.3	0.0
	TRINITY_DN67870_c2_g2_i1	CSLC5	750.6	1896.6	205.1
	TRINITY_DN67870_c2_g2_i2	CSLC5	122.9	325.2	39.5
	TRINITY_DN53435_c1_g1_i22	CSLE6	2.7	22.2	44.0
	TRINITY_DN53435_c1_g1_i4	CSLE6	0.0	34.8	90.5
	TRINITY_DN68740_c0_g1_i19	CSLC12	53.4	0.0	79.5
	TRINITY_DN67808_c1_g1_i8	CSLG3	261.9	47.0	167.7
	cluster_contig12483_	CSLG1	96.7	15.6	65.4
29	TRINITY_DN61387_c1_g1_i7	EXPA8	32.0	85.6	0.8
	TRINITY_DN52980_c0_g1_i3	EXPA2	33.4	132.7	7.1
	TRINITY_DN52980_c0_g1_i2	EXPA2	58.7	195.8	11.6
	TRINITY_DN52980_c0_g1_i1	EXPA2	3.9	64.6	3.4
	TRINITY_DN61387_c1_g1_i4	EXPA8	6.2	217.4	1.0
	TRINITY_DN66245_c1_g1_i1	EXPA8	353.5	3959.7	123.8
	TRINITY_DN61387_c1_g1_i1	EXPA8	191.6	979.1	74.5
	TRINITY_DN53994_c0_g5_i2	EXPA8	73.7	482.9	22.0
	TRINITY_DN66245_c0_g3_i1	EXPA8	109.7	587.3	38.7
	TRINITY_DN61387_c1_g1_i3	EXPA8	370.5	1261.6	155.3
	TRINITY_DN53994_c0_g5_i4	EXPA8	484.1	2408.0	121.9
	cluster_contig39757_	EXPA8	717.7	3442.0	209.8
	TRINITY_DN53994_c0_g5_i3	EXPA8	40.6	136.2	15.1
30	TRINITY_DN62483_c0_g4_i1	XTH23	55.3	10.1	97.4
	cluster_contig31913_	XTH15	99.1	6.1	169.9
	cluster_contig20586_	XTH30	3.1	0.0	28.0
	cluster_contig27198_	XTH23	212.0	98.4	463.3
	cluster_contig31732_	XTH22	99.1	39.9	276.8
	TRINITY_DN54213_c0_g2_i1	XTH7	594.5	216.4	1026.9
	cluster_contig43254_	XTH22	39.6	6.5	87.9
	TRINITY_DN62483_c0_g1_i2	XTH12	331.7	102.2	723.2
	TRINITY_DN62483_c0_g1_i4	XTH12	504.0	239.3	902.7
	TRINITY_DN52754_c7_g1_i1	XTH13	165.6	80.0	318.8
	TRINITY_DN69511_c2_g1_i1	XTH1	0.9	5.6	247.9
	cluster_contig38925_	XTH32	3.7	25.0	320.4
	cluster_contig35350_	XTH2	4.1	36.1	1319.4
	TRINITY_DN63641_c0_g1_i1	XTH2	275.2	191.2	908.7
	TRINITY_DN63641_c0_g1_i2	XTH23	286.0	230.0	1105.1
	cluster_contig28981_	XTH23	134.5	119.8	501.7
	TRINITY_DN59176_c3_g5_i1	XTH22	462.6	421.3	2105.0
	TRINITY_DN61200_c0_g5_i1	XTH5	1365.0	4672.6	807.0
	TRINITY_DN52982_c0_g1_i4	XTH32	13.0	128.2	90.0
	TRINITY_DN52982_c0_g1_i3	XTH32	9.2	111.2	95.4

#### Cell Wall Modification

In flowering plants, cellulose is a para-crystalline array of about two to three dozen (1→4)-β-d-glucan chains and is the main load-bearing polymer of the cell wall. The length, angle, and crystallinity of cellulose microfibrils determine the physical characteristics of the cell wall (Nishiyama, 2009). The cellulose synthase superfamily has been classified into nine cellulose synthase-like (CSL) families and one cellulose synthase (CesA) family. In the transcriptome profile, *CesA1* was pronouncedly upregulated at T1, while *CesA8* and *CSLC5* were activated at T2, suggesting that flower opening and closure requires the participation of newly synthesized polysaccharose (Figure 5B and Table 4). In addition, eight out of 10 *CSLEs* and two *CSLGs* were downregulated at T3 and T2, respectively, implying that the synthesis of cellulose was regulated by different members of CSLs in the flower closure stages, possibly causing a change in the physical characteristics of the cell wall that influence petal movement (Figure 5B and Table 4).

The synthesized cell wall components can be loosened by apoplastic enzymes. Xyloglucan endotransglycosylase/hydrolase (XTH) can cleave and/or rearrange the xyloglucan backbones in plants and have two distinct catalytic activities: xyloglucan endohydrolase (XEH) activity and xyloglucan endotransglycosylase (XET) activity (Rose et al., 2003). Expansins (EXPs) promote cell wall acidification to increase wall extensibility without the lysis of wall polymers (Cosgrove, 2015). The expression levels of *EXPA2* and *EXPA8* were higher at T2 compared to T3 (Figure 5B and Table 4). Conversely, most of the *XTH* homologs showed upregulation at T3 (Figure 5B and Table 4). The distinct responsiveness of *EXPs* and *XTHs* to the two floral closing stages in our study implies that EXP-mediated wall loosening is a key event in flower temporary closure, while the later stage rather relies on XTH-dependent cell wall microfibril rearrangement.

### Gene Regulation of Transcription Factors

Apart from the Aux/IAAs and EFRs, more than 120 unigenes encoding for TFs were also identified in this research (Supplementary Table 2), and these unigenes generally belonged to five families: MYB, WRKY, NAC, Zinc-finger, and Homeobox. The majority of the TFs were upregulated at the T3 stage (Supplementary Table 2), suggesting the underlying role of TFs in the last closure movement of waterlily petals.

### qRT-PCR Analysis

In order to verify the credibility of transcriptome sequencing data, the sequences of 13 unigenes, which were differential expressed between the three flowering stages, were subjected to fluorescent quantitative analysis of qRT-PCR with the designed primers. The RT-qPCR results of 12 unigenes were consistent with those obtained with RNA-Seq method (upregulated or downregulated, Table 5). For example, abundance of TRINITY_DN69808_c4_g1_i1, homolog of Floral homeotic protein PMADS 2, was 3.39-fold higher in T1 stage than T3 stage by qRT-PCR, while it was 4.41-fold upregulated (T1 vs. T3) from RNA-Seq analysis. Except for one transcript (cluster_contig22599_), its regulation in RT-qPCR was opposite to the sequencing.

**TABLE 5:** The qRT-PCR with the differential expressed genes.

Gene ID	Annotation	Relative gene expression by qRT-PCR 2^–ΔΔCT^	RNA-Seq expression Log_2_FC^a^	Comparison
cluster_contig12083_	Jasmonate O-methyltransferase	–1.39 ± 0.42	–2.06	T2 vs. T1
TRINITY_DN54213_c0_g2_i1	Probable xyloglucan endotransglucosylase/hydrolase protein 7	–2.26 ± 0.59	–2.34	T2 vs. T3
cluster_contig12083_	Jasmonate O-methyltransferase	–2.32 ± 0.38	–3.19	
TRINITY_DN52729_c3_g1_i2	Ethylene-responsive transcription factor ERF110	–1.83 ± 0.35	–4.28	
cluster_contig35170_	Aquaporin TIP1-1	+1.64 ± 0.35	+4.08	
TRINITY_DN52238_c0_g1_i2	Probable calcium-binding protein CML45	–2.14 ± 0.06	–4.20	
TRINITY_DN56766_c0_g2_i1	Ribonuclease 3-like protein 2	+1.52 ± 0.20	+2.69	
TRINITY_DN69808_c4_g1_i1	Floral homeotic protein PMADS 2	+3.39 ± 0.52	+4.41	T1 vs. T3
cluster_contig18549_	Indole-3-acetic acid-amido synthetase GH3.3	–2.56 ± 0.38	–4.55	
cluster_contig35170_	Aquaporin TIP1-1	+1.32 ± 0.35	+3.74	
cluster_contig14101_	Indole-3-acetic acid-amido synthetase GH3.5	+1.93 ± 0.01	+2.40	
cluster_contig14537_	Cellulose synthase-like protein E2	+1.43 ± 0.41	+2.67	
TRINITY_DN55271_c8_g1_i2	Auxin-responsive protein SAUR50	+1.45 ± 0.37	+3.09	
TRINITY_DN52186_c0_g1_i1	Respiratory burst oxidase homolog protein C	–1.81 ± 0.12	–3.52	
cluster_contig22599_	Gibberellin-regulated protein 13	–1.51 ± 0.41	+2.86	

*^*a*^Log_2_FC = log_2_ of fold change.*

## Discussion

It is generally believed that the circadian opening and closure movements of flowers are controlled by the “inner clock,” which regulates many important plant growth processes. The clocks are connected to a light signal, a hormone signal, etc., comprising a complex network that regulates cellular processes. As it is challenging to evaluate the transcriptional changes at every moment during flowering, we selected three typical periods based on the opening and closing characteristics of waterlily, including opening on the second flowering day (T1), closure on the second flowering day, and then closure on the fourth (last) flowering day (T3), to determine the molecular clues leading to the termination of flower movement. Unlike flowers which open only once, waterlily flowers would complete the opening and closure movements before withering. However, it is still not known when the senescence process initiates in waterlily flowers. We focused on the T3 stage as this represents the termination of floral movement, and determining gene expression changes at this stage should help us to understand the senescence process in flowers that exhibit circadian movements.

### Petals Entered the Senescence Processes at Least Since T3 Stage

**FIGURE 6:**
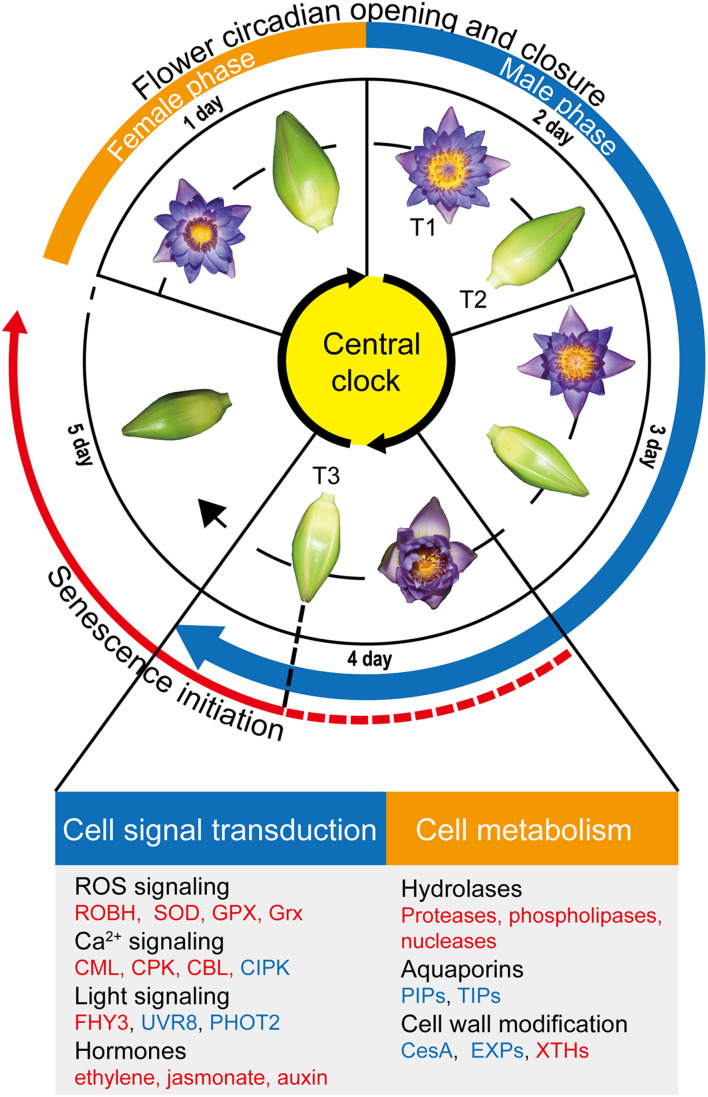
Overview of flower circadian opening and closure and flower senescence regulation. The flowers of *Nymphaea* ‘Blue Bird’ (waterlily) exhibit daily opening and closure movements for four days, followed by no further reopening as well as wilting from the fifth day. waterlily flowers are protogynous; the first blooming day is the female phase, and the male phase begins from the second blooming day and continues to the fourth day. We found that the senescence process was initiated from the last closure stage (T3) in the petals (red solid line), and speculate that the process possibly begins from as early as the start of the fourth day due to the smaller opening size of flowers on the fourth day (red dotted line). Genes involved in cell signal transduction and metabolism were listed below. The upregulated pathways/genes at T3 are marked in red, and the downregulated pathways/genes are marked in blue.

Usually, flower senescence occur in the later flowering stage. Although there are no visible wilting or fading symptoms in petal on the fourth day, the smallest opening angle in the fourth day suggested that senescence process might begin from as early as the start of the fourth day (Figure 6). Furtherly, the transcriptome results indicate that the petals had been undergoing senescence at least since flower closure on the fourth day (Figure 6).

Ca^2+^ is the most versatile second messenger in all eukaryote organisms and is involved in almost every aspect of information processing (Batistič and Kudla, 2012; Edel and Kudla, 2016). In the transcriptome, the majority of genes encode Ca^2+^ sensor proteins including *CPKs, CBLs, CMLs* were induced at T3 stage, suggesting that Ca^2+^ signaling is extensively produced at last flower closure time. Among them, it is reported that *CBL1* and *CBL3* are induced by nitric oxide (NO) and are considered to be the downstream molecules of NO-mediated cut lily senescence (Zhang et al., 2018). The transcriptome showed that *CBL1* and *CBL9* were stimulated at T3, implying that Ca^2+^ signaling possibly mediated the senescence process in waterlily flowers (Figure 3A and Table 1). ROS is thought to play an important signaling role in plant development (Mittler, 2016). Our results showed the gene assigned to ROS-producing and ROS-scavenging were both upregulated at T3 stage, which might cause the ROS levels to fall outside a suitable range and have a detrimental impact on plant health (Mittler, 2016), and the changes in ROS homeostasis possibly disrupted flower opening and closure oscillations, leading to petal senescence.

It is still not known how the light perception signals are transduced to control floral movement and longevity. The flowers of *Eustoma grandiflorum* also possess opening and closure rhythms, the rhythms could be synchronized both by blue and by red light. Especially, flowers opened more rapidly under high blue light intensity irradiation, while similar intensity-dependent effect was not observed for red light (Bai and Kawabata, 2015). Interestingly, our results showed the blue light and UV-B signaling were active at T2 stage, which implied that the blue light/UV-B mediate the petal closure in second day. Phototropin1 (PHOT1), a blue light receptor, declines during leaves senescence in Arabidopsis (Łabuz et al., 2012). Similarly, we found POHT2 was downregulated at T3 stage, which suggested senescence process had occurred in petals and the reaction to blue light was weaken at the last closure stage. In contrast, the red light signaling showed active at T3 stage, we found *FHY3*, a TF that controls Phytochrome A (PHYA) nuclear accumulation and that has been reported to negatively regulate leaf senescence in *Arabidopsis thaliana* (Lin et al., 2008; Tian et al., 2020), was upregulated at T3, implying that the pathways mediated by far-red light photoreceptor PHYA were stimulated upon flower closure on the fourth day, which might regulate the petal senescence process.

Gibberellin and ethylene often promote and inhibit growth of plant, respectively. GA-induced growth and development in plants is modulated by DELLA proteins, which act as repressors of growth (Nakajima et al., 2006). When GA accumulates and binds to the GIBBERELLIN INSENSITIVE DWARF1 (GID1) GA receptors, DELLA proteins are degraded, thereby relieving their growth-restraining effects (Fu et al., 2004). The upregulated GA inactivated enzyme *GA2ox* was found at T3 stage, which might release the DELLA proteins and repress petal growth. While ethylene can accelerate senescence in the ethylene-sensitive flowers, like Petunia, carnation, Ipomoea, and rose (Tripathi and Tuteja, 2007). It is still not clear that whether waterlily flowers are ethylene sensitive or not, which is an important issue. If the waterlily flowers are sensitive to ethylene, the flower ethylene production during the night (flower closure) must be regulated strictly, because the closed flower make a closed environment which will enhance the effect of the gaseous hormone. Interestingly, the ethylene synthesis enzyme *ACO* and *ERF* family members were upregulated at T3, which suggested that the ethylene signal probably regulated the senescence process of petal. Besides, genes involved in the other airborne signal, Methyl jasmonate, were also induced at T3 stage, which might accelerate petal senescence by integrated ethylene signaling (Zhang et al., 2019). So, the two gaseous hormones are worth to study further, as they might directly lead to the senescence process in the closed flower of waterlily. Besides, auxin was proved to be able to mediate the circadian flower opening and closure of waterlily (Ke et al., 2018). In our results, most early auxin response genes including *SAURs, IAAs and GH3* were upregulated at T3 stage, implying the underlying role of auxin signaling in waterlily petal final closure.

Flower senescence is a strictly regulated and involves the expression of different hydrolases that hydrolyze polymers such as proteins, lipids, and nucleic acids (Tripathi and Tuteja, 2007). Among all the proteases, cysteine proteases have been exclusively reported to be involved in petal senescence and are related to the remobilization of nutrients from senescing floral tissues (Shahri and Tahir, 2013). In line with the upregulation of cysteine proteases and aspartyl proteinases during various floral senescence stages in previous reports (Breeze et al., 2004; Jones et al., 2005), *SAGs* and *APs* were upregulated at T3. What’s more, gene assigned to phospholipases, and nucleases were also stimulated at T3. These data indicated that the senescence process entered into the execution phase at the last closure stage (Tripathi and Tuteja, 2007).

### Changes in Water Transportation and Cell Wall Modification Eventually Lead to the Termination of Petal Movements

It has been proved that the opening and closing mechanism of the petals of the waterlily is carried out by the expansion and contraction of the base petal cells (Ke et al., 2018). And the cell expansion is driven by turgor pressure and involves delicately coordinated cycles of acidification and hydration of the cell wall and loosening of cell wall cross-linkages (Wolf et al., 2011). Hence, cell water flow (influx and outflux) and cell wall modification are core components in petal movements. In the transcriptome profiles, genes assigned to Aquaporins (APQs) were regulated differently between the two closure stages. Compared with other APQs, NIPs have lower water permeability and are located in the plasma and intracellular membrane (Maurel, 2007). The results showed that *NIP1-1* was more highly expressed at T2 and T3, suggesting that NIPs play a fundamental water transport role during flower closure (Figure 5B and Table 4). TIPs are located in the tonoplast, and the water permeability of the tonoplast is considered to be much higher than that of the plasma membrane, which results in the rapid osmotic adjustment of the cytoplasm and maintenance of cell turgor pressure (Kapilan et al., 2018). Isoforms of PIP2 are considered to be the main pathway for cell-to-cell water transport (Kaldenhoff and Fischer, 2006). Rh-PIP2-1 was involved in petal expansion in rose, and petal expansion was inhibited in *Rh-PIP2-1*-silenced flowers (Ma et al., 2008). The results showed that the activity level of water flow in petal cells was higher at T2 than at T3, because *TIPs* and *PIPs* were more highly expressed at T2 and were suppressed at T3 (Figure 5B and Table 4). And the petal cells might have lost the turgor pressure necessary for petal movements with the restriction of water transport at T3 stage. Thus, these gene changes in aquaporin density and type might be one of the reasons why the petals no longer open following the last closure. Similarly, in *Hibiscus rosa-sinensis*, genes assigned to APQs were higher expressed in the bud flowers and opening flowers than in the senescent flowers (Trivellini et al., 2016), which suggested the water transportation was reduced during flower senescence. And one research found that the water balance value on the fifth flowering day was significantly lower than that on the fourth day (*P* < 0.05) in waterlily flower (Sheng et al., 2019), which suggested the occurrence of water loss on the fifth day. Further, our results indicated that the decline in water transport capacity appeared as early as the T3 stage. Moreover, the expression of APQs genes could be regulated by hormones such as ethylene and auxin. Ethylene suppressed the expression of *RhPIPs* in petals of rose (Ma et al., 2008; Chen et al., 2013). And auxin repressed the expression of *PgTIP1* in ginseng (*Panax ginseng*) cells (Lin et al., 2007). Our results showed the ethylene and auxin signals were induced at T3, which might mediate petal senescence in part by affecting the expression of APQs.

Genes of the two enzymes, EXPs and XTHs, were induced at T2 and T3, respectively. These results suggested that the way of cell wall modification were different between the two closure stages. In the temporary closure stage (T2), cell wall was modified by EXP acidification. In the final closing stage (T3), the xyloglucan skeleton of the cell wall was rearranged by XTH. Although XTHs could loosen cell wall and were accumulated when flower opening (Van Sandt et al., 2007; Ochiai et al., 2013), upregulation of *XTHs* was also found in senescent flowers (Singh et al., 2011; Trivellini et al., 2016). Moreover, some *XTH* members have been shown to be ethylene responsive during petal senescence process of rose (Singh et al., 2011), which implied the upregulation of *XTHs* in waterlily petals might be related to the elevated ethylene signaling occurred at T3 stage. Further, the XTHs could also promote senescence process, an increase in xyloglucan endotransglucosylase (XET) activity of XTH was found in the petal abscission zones, which might allow for easier accessibility of the wall to other hydrolytic enzymes, thus accelerating abscission (Singh et al., 2011) Overall, these data suggested that cell wall-modifying enzymes mediated cellular expansion and collapse during flower development and aging processes, and the increased expression level of *XTH* at T3 was probable one of the important senescence events that caused the petal cells losing the expansion ability.

Above discussion showed the gene expression changes of water transportation and cell wall modification that occurred at T3 stage might be caused by the regulatory signals in senescence process such as hormones. On the one hand, we speculated that the senescence process had been initiated as early as the start of the fourth day and weakened the opening-closure rhythm (the opening angle was smallest on the fourth day), and finally terminated the rhythm at T3 stage. However, on the other hand, whether the termination of the rhythm could act as a growth signal and in turn promote the aging process? Like the pollination that can trigger senescence in some flowers (van Doorn and Kamdee, 2014). It needs further investigation.

### Transcription Factors Involve in the Petal Senescence Process of Waterlily Flowers

Many TF members belonging to MYB, WRKY, NAC, Zinc-finger, and Homeobox were also stimulated at T3 (Supplementary Table 2). For instance, *MYB44*, whose overexpression delays the senescence of Arabidopsis leaves, and seems to have a homeostatic function of maintaining the growth process under stress conditions (Jaradat et al., 2013), was stimulated at the last closure stage, implying that it might coordinate the petal senescence process in waterlily. Moreover, NAC and WRKY TFs are central regulators in modulating transcription changes during senescence (Kim et al., 2016). Our transcriptome profiles showed that *WRKY24*, *WRKY76*, and *NAC29* were upregulated at T3, which suggested that TFs play an important role in regulating petal senescence during flower closure on the fourth day.

### Termination of Petal Movements May Be Related to the Process of Stamens Maturation

Like most waterlilies, *Nymphaea* ‘Blue Bird’ is a protogynous species, the male phase of the flower is longer than the female phase. Accompanied by anther dehiscence, the stamens gradually opened (matured) from the second day to the fourth day, starting from the outside (Figure 1C). Notably, the fact that all the stamens matured on the fourth day is parallel to the fact that petal movement will no longer occur after closure on the fourth day. It is sensible that flowers need not waste energy to reopen when there is no fresh pollen after the fourth day, which supports the view that the purpose of flower closure might be to prevent the dispersal of pollen with reduced viability (van Doorn and van Meeteren, 2003; Franchi et al., 2014). Thus, we speculate that the termination of petal movements may be related to the stamens mature. Next, if the relation really exists, will the stamens directly influence petal movements? Or do the stamens affect petal senescence first, and then cause petal movement to cease? The latter hypothesis sound more sensible because flower senescence (wilt) was proven to be promoted by some developmental signals, such as pollination. In ornamental plants such as orchids, roses, and petunia, petal senescence occurs shortly after pollination as a result of ethylene generation (Rogers, 2006, 2013). However, more experiments are needed, such as removing stamens from flowers to verify the effect of stamens on petals during the flowering process of waterlilies.

## Data Availability Statement

The datasets presented in this study can be found in online repositories. The names of the repository/repositories and accession number(s) can be found below: https://www.ncbi.nlm.nih.gov/bioproject/PRJNA673637.

## Author Contributions

ZL and YZ designed the experiments. WZ, YC, and SH performed the records of flower movements. PW performed the RNA-Seq. ZL, PW, JW, DT, and JN analyzed the data. ZL visualized the data and drafted the manuscript that was critically revised by DT, YZ, and XS. YZ had the overall responsibility for experimental design and manuscript preparation. All authors read and approved the final manuscript.

## Conflict of Interest

The authors declare that the research was conducted in the absence of any commercial or financial relationships that could be construed as a potential conflict of interest.

## Publisher’s Note

All claims expressed in this article are solely those of the authors and do not necessarily represent those of their affiliated organizations, or those of the publisher, the editors and the reviewers. Any product that may be evaluated in this article, or claim that may be made by its manufacturer, is not guaranteed or endorsed by the publisher.

## Footnotes

1
https://www.internationalwaterlilycollection.com/?project~=~nymphaea-blue-birdr

2
http://www.genome.jp/kegg/

## Supplementary Material

Supplementary Figure 1 Photos of flower opening and closure at 30-min intervals during five flowering days.

Supplementary Figure 2 Volcano plots of up/down differential expressed genes (DEGs).

Supplementary Figure 3 Maps of GO enrichment and KEGG enrichment of differential expressed genes (DEGs).

Supplementary Table 1 Designed primers for qRT-PCR.

Supplementary Table 2 Transcriptome profile of transcriptional factors.
