# Supplementary material for: Transcriptome Analysis Reveals the Senescence Process Controlling the Flower Opening and Closure Rhythm in the Waterlilies (Nymphaea L.)
Source: Front Plant Sci. 2021 Oct 4;12:701633. doi: 10.3389/fpls.2021.701633 (PMC8521120; doi:10.3389/fpls.2021.701633)

Fig. S1. Photos of flower opening and closure at 30-min intervals during five flowering days. The opening-closure process of waterlily flower in each flowering day was shown in following periods: 9:30–15:30 in the first day, 7:00–15:30 in the second day to the fourth day , and 7:00–13:30 in the fifth day.

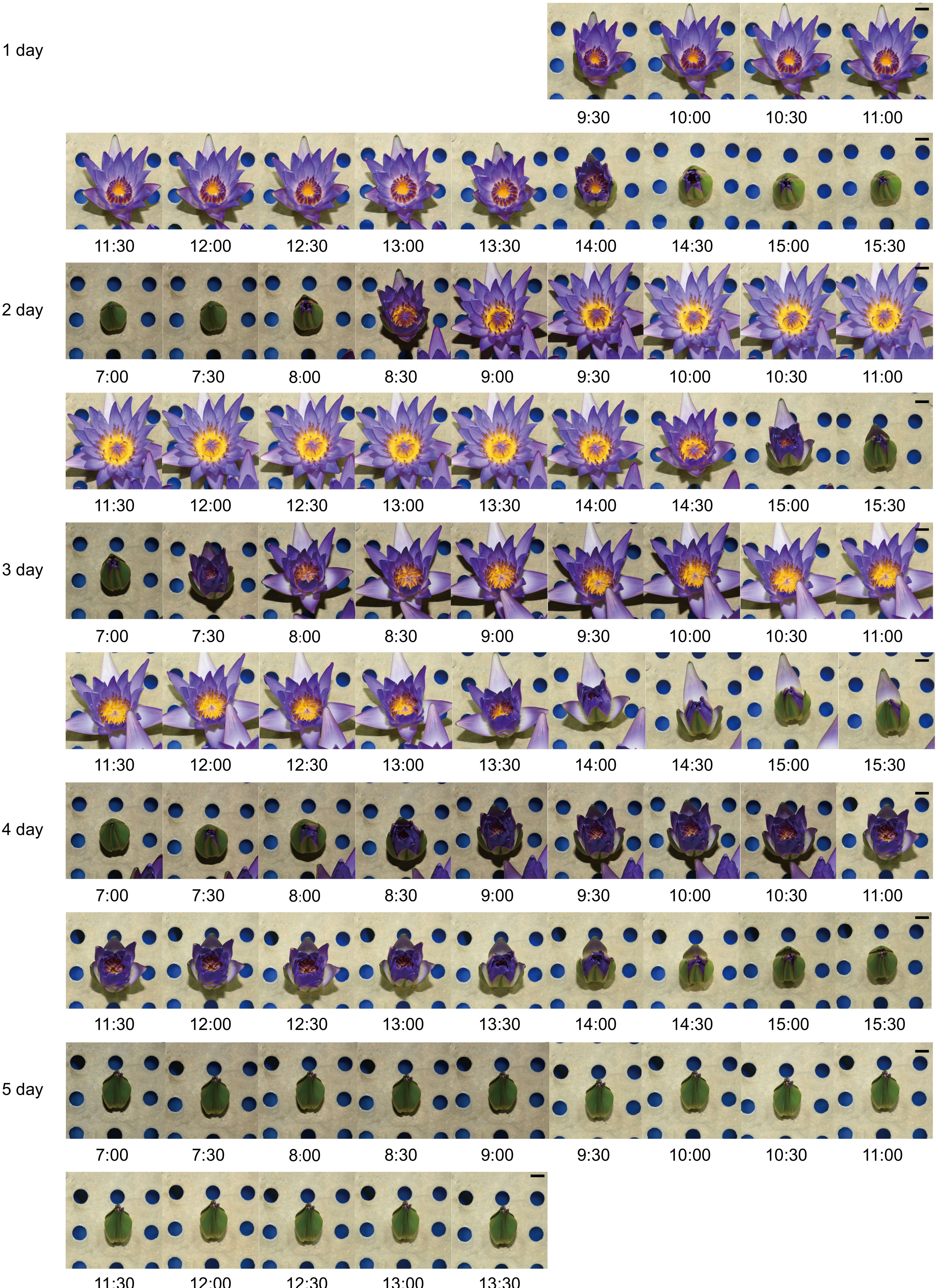

Supplement: Supplementary Figure 1 — Photos of flower opening and closure at 30-min intervals during five flowering days. [file Image_1.pdf]
