# Supplementary figures and images for: Transcriptome Analysis Reveals the Senescence Process Controlling the Flower Opening and Closure Rhythm in the Waterlilies (Nymphaea L.)
Source: Front Plant Sci. 2021 Oct 4;12:701633. doi: 10.3389/fpls.2021.701633 (PMC8521120; doi:10.3389/fpls.2021.701633)

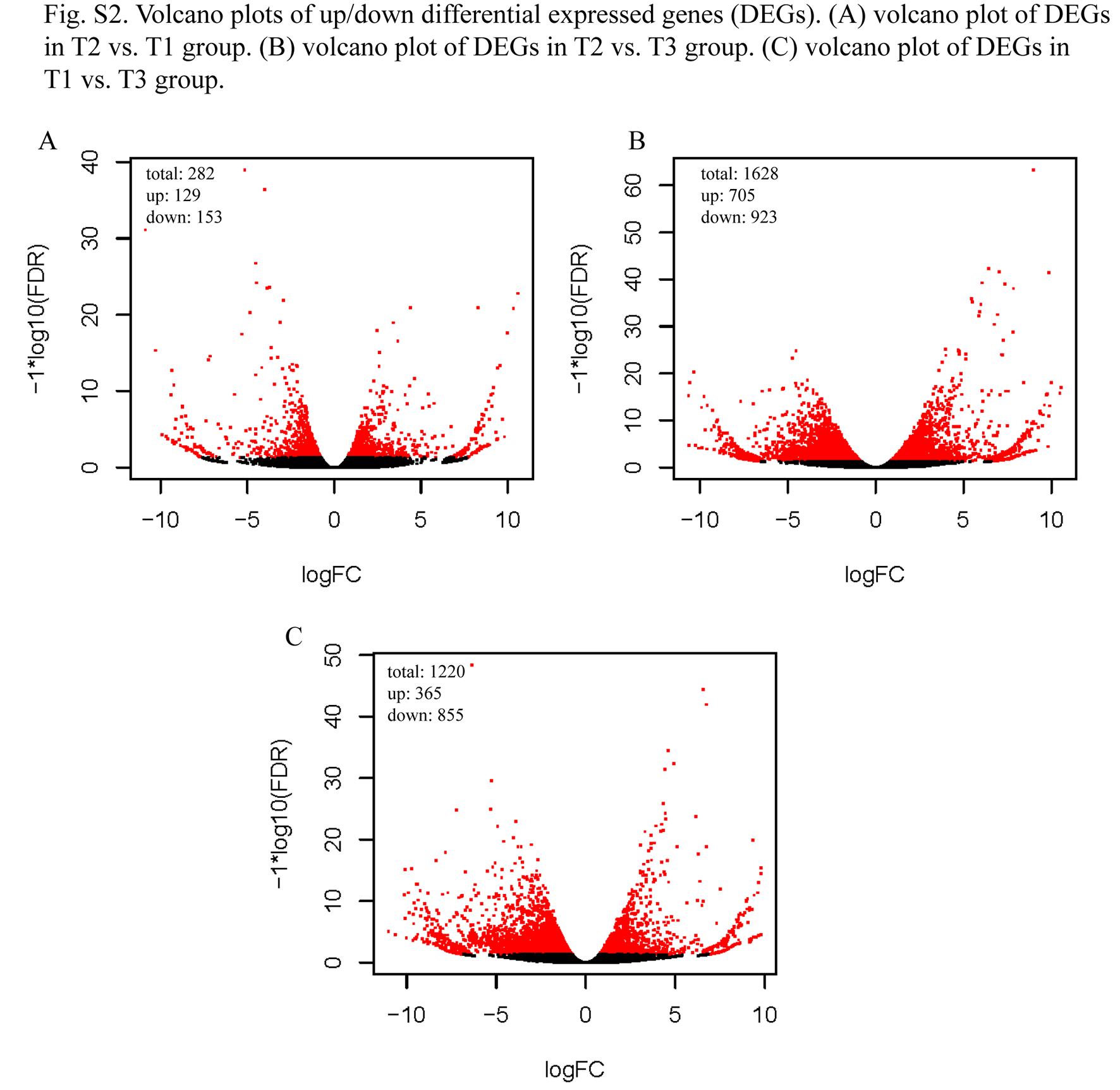

Supplement: Supplementary Figure 2 — Volcano plots of up/down differential expressed genes (DEGs). [file Image_2.TIF]
