# Supplementary material for: Transcriptome Analysis Reveals the Senescence Process Controlling the Flower Opening and Closure Rhythm in the Waterlilies (Nymphaea L.)
Source: Front Plant Sci. 2021 Oct 4;12:701633. doi: 10.3389/fpls.2021.701633 (PMC8521120; doi:10.3389/fpls.2021.701633)
Supplement: Supplementary Figure 3 — Maps of GO enrichment and KEGG enrichment of differential expressed genes (DEGs). [file Image_3.PDF]

Fig. S3. Maps of GO enrichment and KEGG pathway enrichment of the differential expressed genes (DEGs). (**A**) GO items of DEGs in T2 vs. T1 group. (**B)** Enriched KEGG pathways of DEGs in T2 vs. T1 group. **(C)** GO items of DEGs in T2 vs. T3 group. (**D)** Enriched KEGG pathways of DEGs in T2 vs. T3 group. (**E)** GO items of DEGs in T1 vs. T3 group. (**F)** Enriched KEGG pathways of DEGs in T1 vs. T3 group.

A

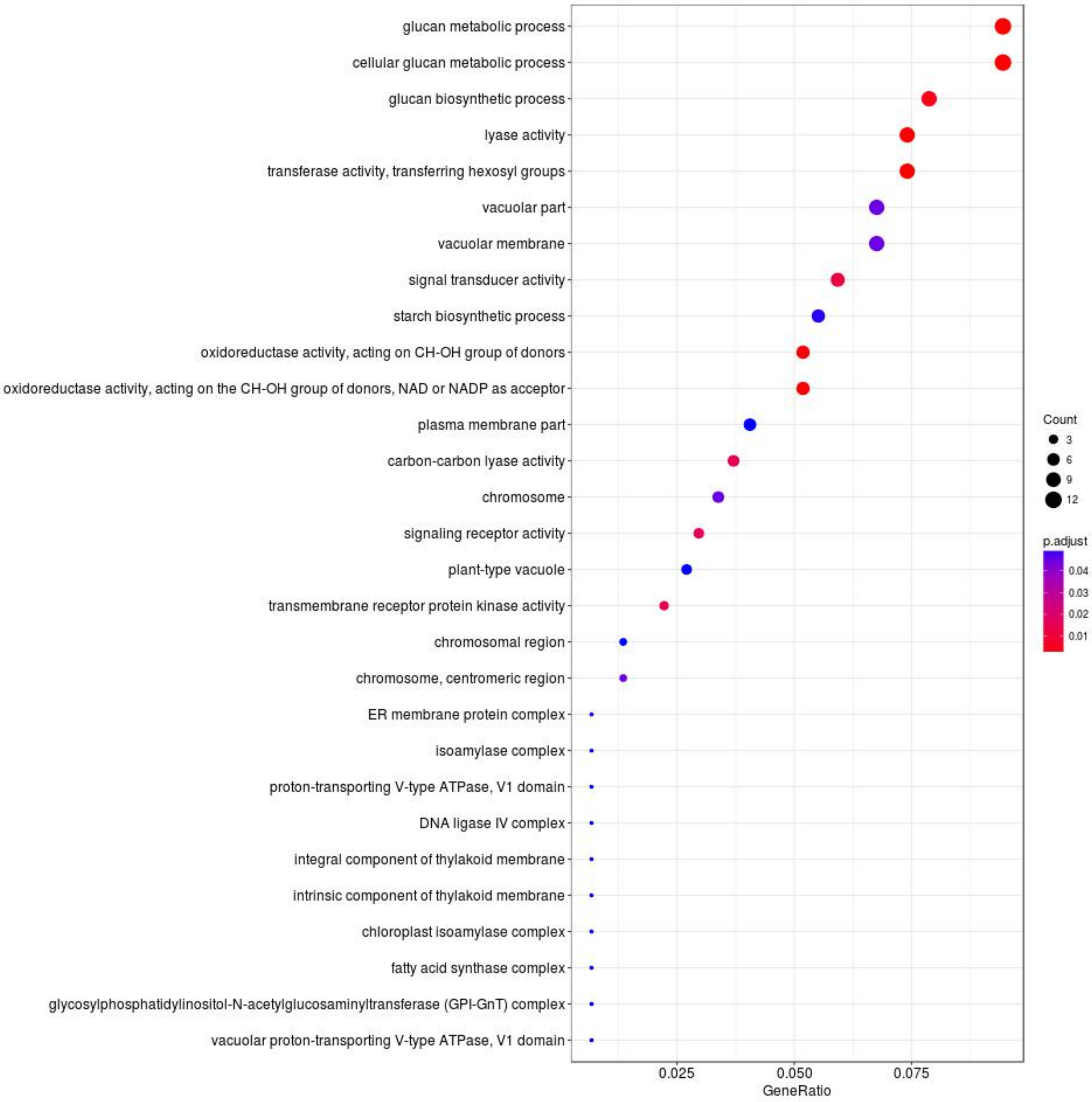

B

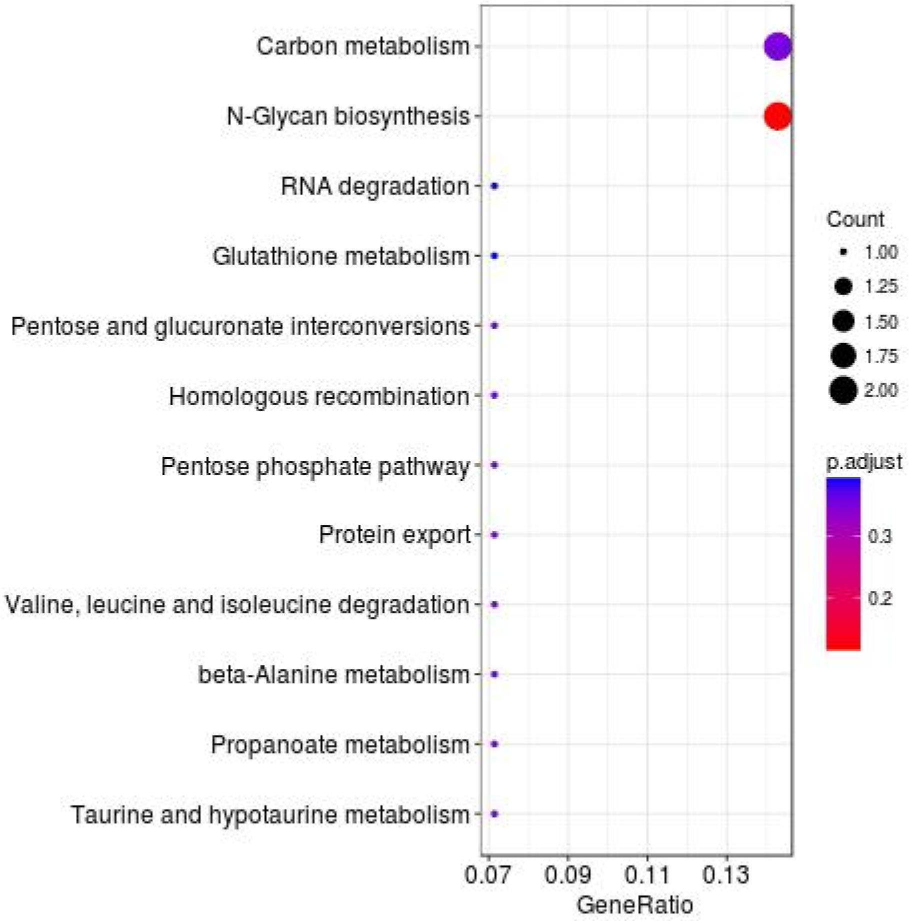

Carbon metabolism
N-Glycan biosynthesis
RNA degradation
Glutathione metabolism
Pentose and glucuronate interconversions
Homologous recombination
Pentose phosphate pathway
Protein export
Valine, leucine and isoleucine degradation
beta-Alanine metabolism
Propanoate metabolism
Taurine and hypotaurine metabolism
0.07
0.09
0.11
0.13
GeneRatio
Count
1.00
1.25
1.50
1.75
2.00
p.adjust
0.3
0.2

C

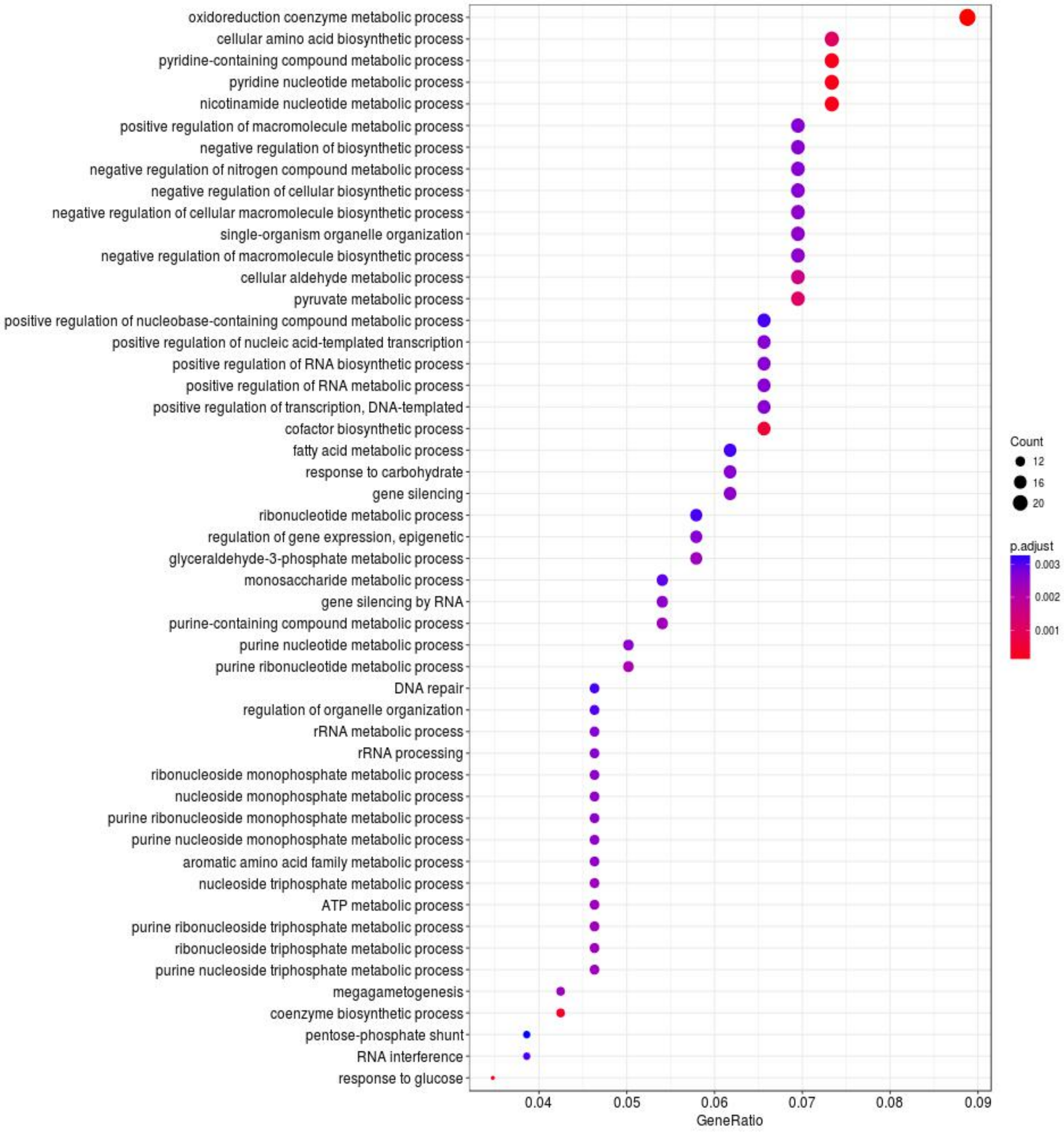

oxidoreduction coenzyme metabolic process
cellular amino acid biosynthetic process
pyridine-containing compound metabolic process
pyridine nucleotide metabolic process
nicotinamide nucleotide metabolic process
positive regulation of macromolecule metabolic process
negative regulation of biosynthetic process
negative regulation of nitrogen compound metabolic process
negative regulation of cellular biosynthetic process
negative regulation of cellular macromolecule biosynthetic process
single-organism organelle organization
negative regulation of macromolecule biosynthetic process
cellular aldehyde metabolic process
pyruvate metabolic process
positive regulation of nucleobase-containing compound metabolic process
positive regulation of nucleic acid-templated transcription
positive regulation of RNA biosynthetic process
positive regulation of RNA metabolic process
positive regulation of transcription, DNA-templated
cofactor biosynthetic process
fatty acid metabolic process
response to carbohydrate
gene silencing
ribonucleotide metabolic process
regulation of gene expression, epigenetic
glyceraldehyde-3-phosphate metabolic process
monosaccharide metabolic process
gene silencing by RNA
purine-containing compound metabolic process
purine nucleotide metabolic process
purine ribonucleotide metabolic process
DNA repair
regulation of organelle organization
rRNA metabolic process
rRNA processing
ribonucleoside monophosphate metabolic process
nucleoside monophosphate metabolic process
purine ribonucleoside monophosphate metabolic process
purine nucleoside monophosphate metabolic process
aromatic amino acid family metabolic process
nucleoside triphosphate metabolic process
ATP metabolic process
purine ribonucleoside triphosphate metabolic process
ribonucleoside triphosphate metabolic process
purine nucleoside triphosphate metabolic process
megagametogenesis
coenzyme biosynthetic process
pentose-phosphate shunt
RNA interference
response to glucose
0.04
0.05
0.06
0.07
0.08
0.09
GeneRatio
Count
12
16
20
p.adjust
0.003
0.002
0.001

D

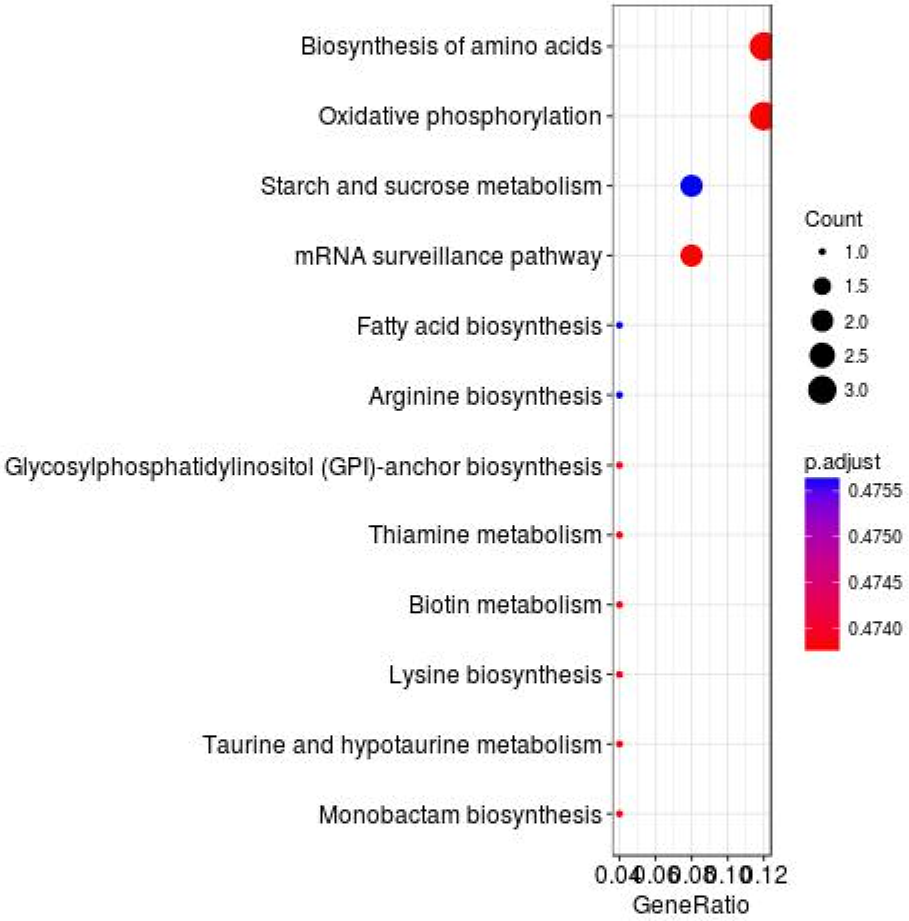

Biosynthesis of amino acids
Oxidative phosphorylation
Starch and sucrose metabolism
mRNA surveillance pathway
Fatty acid biosynthesis
Arginine biosynthesis
Glycosylphosphatidylinositol (GPI)-anchor biosynthesis
Thiamine metabolism
Biotin metabolism
Lysine biosynthesis
Taurine and hypotaurine metabolism
Monobactam biosynthesis
0.04
0.06
0.08
0.10
0.12
GeneRatio
Count
1.0
1.5
2.0
2.5
3.0
p.adjust
0.4755
0.4750
0.4745
0.4740

E

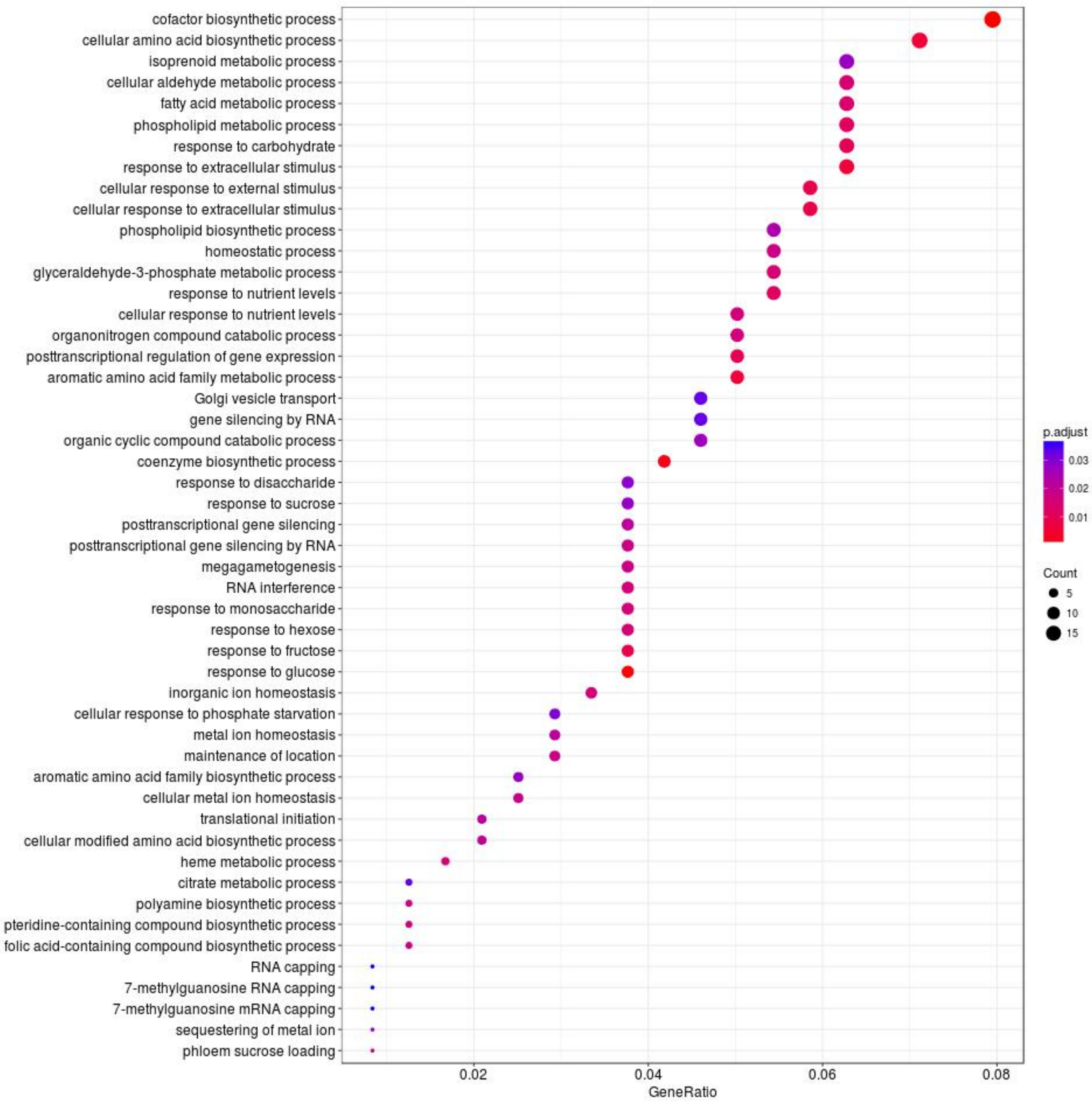
cofactor biosynthetic process
cellular amino acid biosynthetic process
isoprenoid metabolic process
cellular aldehyde metabolic process
fatty acid metabolic process
phospholipid metabolic process
response to carbohydrate
response to extracellular stimulus
cellular response to external stimulus
cellular response to extracellular stimulus
phospholipid biosynthetic process
homeostatic process
glyceraldehyde-3-phosphate metabolic process
response to nutrient levels
cellular response to nutrient levels
organonitrogen compound catabolic process
posttranscriptional regulation of gene expression
aromatic amino acid family metabolic process
Golgi vesicle transport
gene silencing by RNA
organic cyclic compound catabolic process
coenzyme biosynthetic process
response to disaccharide
response to sucrose
posttranscriptional gene silencing
posttranscriptional gene silencing by RNA
megagametogenesis
RNA interference
response to monosaccharide
response to hexose
response to fructose
response to glucose
inorganic ion homeostasis
cellular response to phosphate starvation
metal ion homeostasis
maintenance of location
aromatic amino acid family biosynthetic process
cellular metal ion homeostasis
translational initiation
cellular modified amino acid biosynthetic process
heme metabolic process
citrate metabolic process
polyamine biosynthetic process
pteridine-containing compound biosynthetic process
folic acid-containing compound biosynthetic process
RNA capping
7-methylguanosine RNA capping
7-methylguanosine mRNA capping
sequestering of metal ion
phloem sucrose loading
0.02
0.04
0.06
0.08
GeneRatio
p.adjust
0.03
0.02
0.01
Count
5
10
15

F

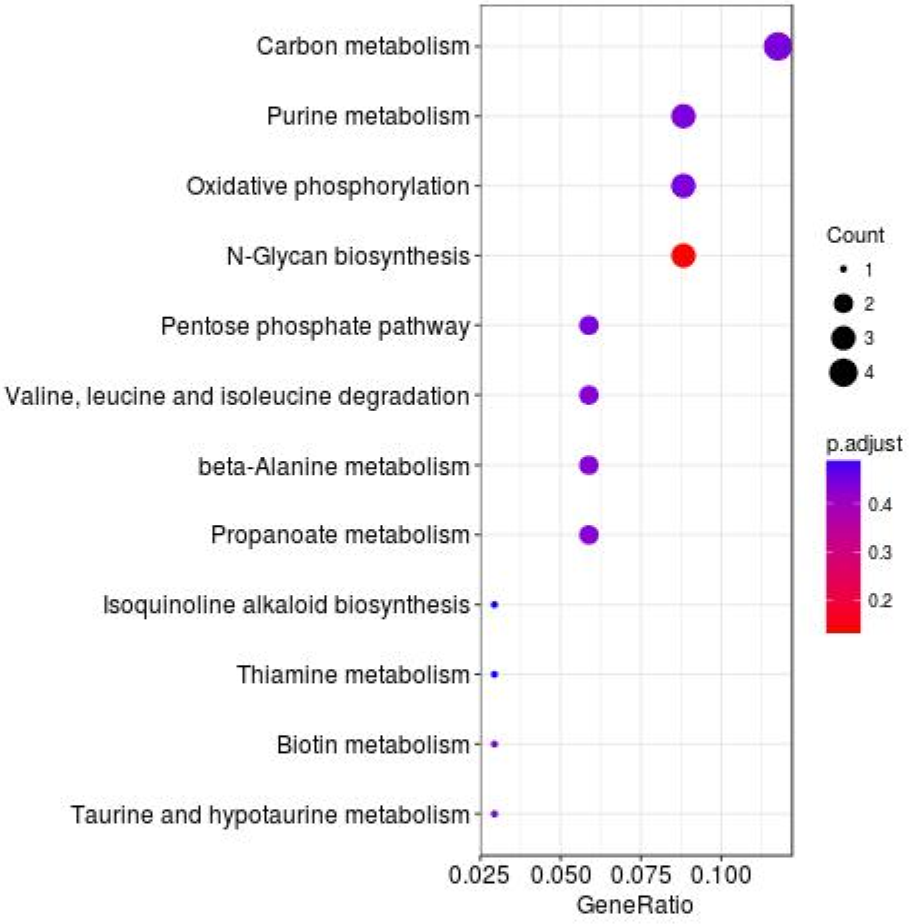
Carbon metabolism
Purine metabolism
Oxidative phosphorylation
N-Glycan biosynthesis
Pentose phosphate pathway
Valine, leucine and isoleucine degradation
beta-Alanine metabolism
Propanoate metabolism
Isoquinoline alkaloid biosynthesis
Thiamine metabolism
Biotin metabolism
Taurine and hypotaurine metabolism
0.025
0.050
0.075
0.100
GeneRatio
Count
1
2
3
4
p.adjust
0.4
0.3
0.2
