# Supplementary material for: Transcriptome Analysis Reveals the Senescence Process Controlling the Flower Opening and Closure Rhythm in the Waterlilies (Nymphaea L.)
Source: Front Plant Sci. 2021 Oct 4;12:701633. doi: 10.3389/fpls.2021.701633 (PMC8521120; doi:10.3389/fpls.2021.701633)
Supplement: Supplementary Table 1 — Designed primers for qRT-PCR. [file Table_1.DOCX]

**Table S1. Designed primers for qRT-PCR**

| Unigene id | Gene name | Forward primer sequence (5'–3') | Reverse primer sequence (5'–3') |
| --- | --- | --- | --- |
| cluster_contig12083_ | Jasmonate O-methyltransferase | GTGGGGATTGAAAGGGGGA | GAACGCAGTTGGCGGAGG |
| TRINITY_DN54213_c0_g2_i1 | Probable xyloglucan endotransglucosylase/hydrolase protein 7 | GGTGGAACGGAGGAGGGT | CTGGGGGAGGGACAGGAT |
| TRINITY_DN52729_c3_g1_i2 | Ethylene-responsive transcription factor ERF110 | GTTCCTAAATGAGCCCGATGA | AAATGCCCAATGCCGTTC |
| cluster_contig35170_ | Aquaporin TIP1-1 | GCCTGCCTCCTCCTCAAGT | GGTGCCGAGCGTTCCC |
| TRINITY_DN52238_c0_g1_i2 | Probable calcium-binding protein CML45 | CCCGAAGATGTGACGGTATG | GGTGGACAAGTGCGAGAGAA |
| TRINITY_DN56766_c0_g2_i1 | Ribonuclease 3-like protein 2 | CGCACCGTTTTCTTCCTTCTT | CCACCGTTCCCACCACTTACT |
| TRINITY_DN69808_c4_g1_i1 | Floral homeotic protein PMADS 2 | GAAGCAGACCGAATGCCTAAA | AACCCAACTGGCTGAACCCT |
| cluster_contig18549_ | Indole-3-acetic acid-amido synthetase GH3.3 | AGGCGAGGCTGAAGTGTGAG | AAGGGTGGTGAAGAGTGGGA |
| cluster_contig14537_ | Cellulose synthase-like protein E2 | GCAGCCATCTTCCTGTCTACC | CCTGTTCCGCACCTCACCT |
| TRINITY_DN55271_c8_g1_i2 | Auxin-responsive protein SAUR50 | CCATCTCGCTTCTTAGCCACC | CTCTTCGCAAGGAATCGTCAA |
| TRINITY_DN52186_c0_g1_i1 | Respiratory burst oxidase homolog protein C | AGGAGAGCAACCCCATCAAG | GCAGACGCAGTAGCCCATAA |
| cluster_contig14101_ | Indole-3-acetic acid-amido synthetase GH3.5 | TTGCGATGAAGGAGAAGGAGG | GTTGGCATTGGTGGAGACGAT |
| cluster_contig22599_ | Gibberellin-regulated protein 13 | CTGACCAAACTACCCCTTATCCAA | CCACACACTCACCTCCCAACT |
|  | *Act11* | ATGTGGCACTGGACTATGAGC | AGAGTTGTAAGTGGTTTCGTGAAT |
